# Mechanistic constraints on the trade-off between photosynthesis and respiration in response to warming

**DOI:** 10.1126/sciadv.adh8043

**Published:** 2023-09-01

**Authors:** Suzana G. Leles, Naomi M. Levine

**Affiliations:** Department of Marine and Environmental Biology, University of Southern California, Los Angeles, CA, USA.

## Abstract

Phytoplankton are responsible for half of all oxygen production and drive the ocean carbon cycle. Metabolic theory predicts that increasing global temperatures will cause phytoplankton to become more heterotrophic and smaller. Here, we uncover the metabolic trade-offs between cellular space, energy, and stress management driving phytoplankton thermal acclimation and how these might be overcome through evolutionary adaptation. We show that the observed relationships between traits such as chlorophyll, lipid content, C:N, and size can be predicted on the basis of the metabolic demands of the cell, the thermal dependency of transporters, and changes in membrane lipids. We suggest that many of the observed relationships are not fixed physiological constraints but rather can be altered through adaptation. For example, the evolution of lipid metabolism can favor larger cells with higher lipid content to mitigate oxidative stress. These results have implications for rates of carbon sequestration and export in a warmer ocean.

## Teaser

Photosynthetic microbes might defy current trends to become larger and grow faster in a warmer ocean.

## INTRODUCTION

Ocean temperatures are increasing at unprecedented rates due to anthropogenic increases in carbon dioxide in the atmosphere (*1*). These rising temperatures have the potential to substantially alter both rates of carbon cycling in the ocean and marine ecosystem dynamics (*2*–*4*). Here, we focus on the response of phytoplankton to increasing temperatures as phytoplankton drive carbon cycling in the ocean, contribute half of all oxygen production, and form the base of the marine food web (*5*, *6*).

Phytoplankton have a classic unimodal response of growth rate to temperature (*7*). This response can be broken down into several competing processes. As temperatures increase, cellular metabolism speeds up as enzyme kinetics increase due to more collisions between the substrate and the active site of the enzyme (*8*, *9*). This results in faster rates of carbon fixation and respiration (*10*). In addition, warmer temperatures increase rates of protein denaturation (*9*). Overall, respiration rates have been shown to increase more rapidly than carbon fixation with increasing temperature (*10*). However, the specific metabolic constraints driving this relationship is unclear.

Understanding the fundamental molecular and energetic trade-offs that underlie classic trait relationships is critical for predicting how phytoplankton will adapt to a warming ocean. Conventionally, predicted responses have been based on present-day trait correlations derived from empirical evidence. For example, previous work has used these correlations to predict that body size will decrease (*3*, *11*) and ecosystems will become increasingly heterotrophic in response to warming (*2*, *6*). However, we lack a fundamental understanding of the molecular mechanisms behind these two hypothesized responses and thus an understanding of how robust future predictions are on the time scales of anthropologically driven climate change.

The relationships between traits and how traits change as a function of temperature can be altered through adaptation. Here, we determine which of these relationships can be altered to overcome environmental stress given the fundamental constraints on energy and space that a cell must contend with. This is motivated by recent work that showed that some trait correlations (e.g., between metabolism and size) can be altered through adaptation (*12*). An experimental evolution study found that phytoplankton evolved larger cell sizes and higher growth rates due to warming (*13*), opposed to the expected smaller cell sizes (*14*).

By leveraging tools from thermodynamics, resource allocation theory, and trait ecology, we developed a proteome model for a generic phytoplankton cell to investigate the mechanistic constraints on photosynthesis and respiration due to warming. The model optimizes proteome allocation and cell size to maximize phytoplankton growth under different temperatures. We use this model to provide mechanistic insights into the trade-offs between respiration, cellular space, and environmental stress management that limit the metabolic choices of phytoplankton acclimation under temperature stress and how these might be overcome through adaptive change. Our results suggest that phytoplankton acclimate to warming by switching the main respiratory pathway from glycolysis to lipid degradation due to a trade-off based on proteomic cost, energy yield, and space constraints. In addition, decreases in cell size are driven by the thermal dependency of nutrient uptake and by the increased saturation of membrane lipids. We suggest that the relationship between size and temperature can be overcome through changes in lipid metabolism (e.g., increasing lipid content to mitigate oxidative stress). Thus, under certain environmental conditions, phytoplankton might adapt to warming by evolving larger cell sizes with faster growth rates. This is consistent with experimental data that found evidence for higher lipid content (*15*, *16*) and rapid evolution of genes associated with redox homeostasis and oxidative stress (*13*) among warm-evolved phytoplankton. Understanding the hard and soft constraints that drive phytoplankton acclimation and adaptation will allow us to better predict their impact in the carbon cycling and trophic efficiency as the climate changes.

## RESULTS

### A proteome model for a phytoplankton cell

**Fig. 1.**
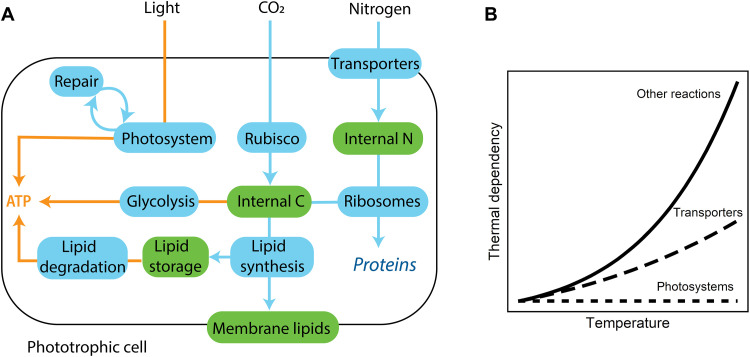
The single-cell proteome model for phytoplankton. (**A**) Blue pools indicate proteins, and green pools indicate other macromolecules. Arrows indicate one-way reactions with blue arrows indicating the production of a given macromolecule catalyzed by a given protein and orange arrows indicating energy production fluxes. The model optimizes the relative investment in the different proteins. (**B**) The model assumes that photosystems are temperature independent and that the thermal dependency of nutrient transporters is lower than that of other reactions (see the “A proteome model for a phytoplankton cell” section). Thermal dependency (unitless) is defined as the temperature impact on the reaction rate (γ_Ta_; Eq. 4).

An illustrative schematic of the single-cell proteome allocation model for phytoplankton thermal responses is shown in Fig. 1A. The model is built as a constrained optimization problem that maximizes the steady-state growth rate of a generic photosynthetic cell given a set of environmental conditions. Specifically, cellular metabolisms are represented by a system of ordinary differential equations, which are solved to estimate the daytime proteome, macromolecular concentrations, and surface area–to–volume ratio of a photosynthetic cell given external temperature, light, and inorganic nutrients (output variables are listed in table S1). Here, we focus on shifts in temperature and so assume that the cell is growing under steady-state ideal light and nutrient conditions. While we do not simulate diurnal cycles, the cell must generate sufficient protein pools and stored carbon to fuel respiration during the night (see the “Energy metabolism” section in Materials and Methods). Resources are allocated to different protein pools (shown in blue), which regulate the production and consumption of other macromolecules (shown in green).

Photosystems convert light energy into chemical energy. The conversion of inorganic carbon into a generic internal carbon pool (akin to carbohydrates) is mediated by rubisco activity. Nitrogen transporters located in the cell membrane bring inorganic nitrogen into the cell. The internal pools of carbon and nitrogen are used by ribosomes to synthesize proteins, including the ribosomes themselves. The internal carbon pool is also used for lipid synthesis and to fuel dark respiration through glycolysis. The lipid synthesis pathway is used both to build membrane lipids and for producing stored lipids. Stored lipids can be used to fuel dark respiration (*17*) through the lipid degradation pathway. Because our model optimizes for the proteome of the cell in a steady-state environment, glycolysis and lipid degradation are mutually exclusive; the optimal cell exclusively uses the more efficient pathway given a specific temperature. In the model, stored lipids can also be used to minimize heat damage (Eqs. 21 and 22). Previous work has demonstrated that phytoplankton store fatty acids as a result of temperature stress (*15*, *18*, *19*) and that these fatty acids can play a role in mitigating heat stress in both phytoplankton (*18*) and plants (*20*, *21*). In addition to sequestering reactive oxygen species (ROS), lipid droplets also regulate membrane composition by removing unsaturated lipids at elevated temperatures (*18*, *21*, *22*). Cells also use antioxidant enzymes to mitigate the impact of ROS. We also explore the impact of using this alternative metabolic pathway for oxidative stress and show similar results to the baseline model (Supplementary Text and see Discussion).

Cell size is determined by a trade-off between membrane real estate for nitrogen transporters (higher surface area–to–volume ratio) and biovolume for internal macromolecules and organelles (lower surface area–to–volume ratio). The surface area of the cell is equal to the area required for the nitrogen transporters and the membrane lipids that are required for membrane integrity (Eqs. 37 to 39). The volume of the cell is constrained by a maximum allowable intracellular density of macromolecules (proteins, storage pools, internal pools of nitrogen and carbon, and ribosomes; see Eqs. 41 to 43). Thus, if the cell “needs” more intracellular macromolecules to grow optimally, it must get bigger (larger volume to accommodate these intracellular components). Lipids and chloroplasts also affect cell size by occupying intracellular space and thus decreasing the volume available for other intracellular molecules (Eqs. 41 to 43). We assume lipids form droplets that take up space calculated using the measured volume of a triglyceride molecule. The model can consider cells of different shapes, but here, we assumed a spherical cell for simplicity.

Warming accelerates all enzymatic reactions in the model, except for photochemistry (Fig. 1B) because photon capture is temperature independent (*23*). Because rubisco and thus carbon fixation are temperature dependent, photochemistry and carbon fixation are modeled separately. The model can impose different thermal dependencies for different reactions (e.g., Fig. 1B). We explore the sensitivity of these thermal dependencies in the “Changes in cell size due to warming” section and in the Supplementary Materials.

The model also accounts for the fact that the lipid per unit area of membrane increases with temperature (Eq. 27). This results from the switch from unsaturated to saturated lipids at warmer temperatures (*24*). Because saturated lipids pack more easily in the membrane due to the lack of kinked hydrocarbon chains, the cell requires a greater concentration of membrane lipids to maintain membrane integrity at higher temperatures.

Last, the model assumes that protein damage increases with increasing temperature (Eqs. 19 and 20). Although intracellular damage due to elevated temperatures is likely to affect all proteins, photosystems are particularly vulnerable because heat damage is commonly accompanied by light stress and photosystem II (and the D1 protein in particular) is closest to the site of ROS formation (*18*, *25*). Thus, we assume that warming results in increased damage to photosystems and thus requires repair by specialized proteins (Eqs. 23 and 24), following Mairet *et al.* (*26*). A version of the model where all protein pools are damaged at high temperatures and require repair is provided in the Supplementary Materials; this model showed qualitatively similar results. The model and parameters are fully defined in the Supplementary Materials (tables S1 to S3).

The model represents a generic phytoplankton cell that must perform basic phototrophic metabolisms. This gives us insight into the universal energetic and space constraints that a phytoplankton cell faces as temperatures increase (“hard” constraints or trade-offs). We also use the model to explore different metabolic strategies that a cell might use to mitigate stress (e.g., different energy sources for dark respiration). This framework can be expanded to compare different metabolic strategies used by different functional groups. For example, the cellular metabolisms represented in the model could be developed to capture diatom-specific metabolisms or altered to include mixotrophy.

Below, we compare our model against previously published physiological, transcriptomic, proteomic, and metabolomic (‘omics) datasets obtained for cultured isolates. While physiological data cover different temperatures across the thermal growth curves, ‘omics datasets typically compare metabolic shifts between two focused temperatures. Therefore, we compared our model against ‘omics datasets qualitatively by describing the direction of change for a given metabolic process (Table 1).

**Table 1.** Comparison between modeled and observed metabolic shifts. Thermal acclimation responses are shown where the arrows correspond to increases (↑) or decreases (↓) when temperatures are increased. Note that observed shifts in investment in nitrogen transporters come from experiments conducted at or below the optimal growth temperature.

Variable	Model	Observation	Data type and reference
Investment in photosystems	↑	↑	Transcriptomics (*27*); proteomics (*19*)
Investment in rubisco	↓	↓	Proteomics (*18*); transcriptomics (*27*)
Investment in transporters	↑	↑	Proteomics (*19*, *30*)
Investment in repair proteins	↑	↑	Transcriptomics (*27*); proteomics (*18*, *19*)
Investment in ribosomes	↓	↓	Transcriptomics (*27*)
Investment in glycolysis	↓	↓	Transcriptomics (*29*); proteomics (*30*)
Investment in lipid degradation	↑	↑	Transcriptomics (*29*)
Synthesis of saturated lipids	↑	↑	Transcriptomics (*27*); proteomics (*18*, *19*)
Concentration of ribosomes	↓	↓	Total RNA (*28*); transcriptomics (*27*); proteomics (*19*)
		↑	Total RNA (*13*, *29*)
Total protein content	↑	↑	Total protein content (*13*, *29*, *30*)
Stored lipids (fatty acids)	↑	↑	Fatty acid content (*15*, *18*); metabolomics (*19*)

### Model comparison against physiological data

**Fig. 2.**
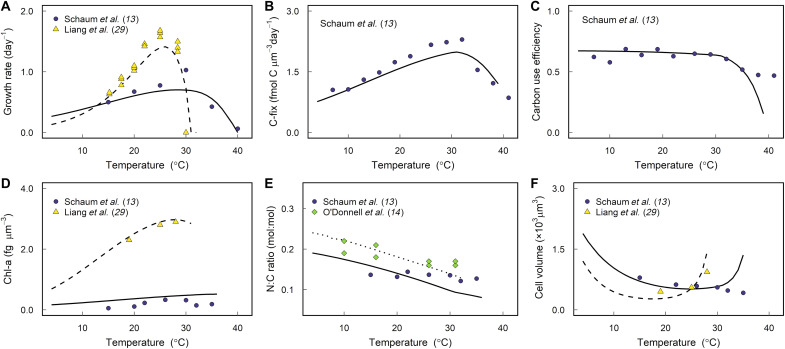
Model validation for thermal acclimation. Comparison between model (lines) and experimental data (symbols) for diatoms acclimated to different temperatures. Solid lines correspond to the model simulation calibrated to data from *T. pseudonana* CCMP1335 (*13*), dashed lines correspond to *Chaetoceros* sp. CCMP160 (*29*), and dotted lines correspond to *T. pseudonana* CCMP1335 (table S3) (*14*). Here, we assume that the cell can only invest in glycolysis to fuel dark respiration, but model validation was also performed assuming that the cell can only invest in lipid degradation (fig. S1). (**A**) Growth rate, (**B**) carbon fixation rate (C-fix), (**C**) carbon use efficiency (unitless and defined as 1 − respiration/photosynthesis where respiration and photosynthesis have units of fmol C μm^−3^ day^−1^), (**D**) chlorophyll-a per unit of biovolume, (**E**) nitrogen to carbon quota of the cell, and (**F**) cell volume.

Our proteome optimization model of a phytoplankton cell reproduces observed trait values and trends as a function of temperature for both large (i.e., diatoms; Fig. 2 and fig. S1) and small (i.e., the cyanobacteria *Prochlorococcus*; fig. S2) phytoplankton. Hereafter, we focus on the model results validated with the diatom data because it allows us to explore the differences between acclimated and adaptive responses using the results from a diatom experimental evolution study (*13*) (see the “Drivers of thermal adaptation” section). The model simulations for small phytoplankton are shown in fig. S2 and show the same energetic and space trade-offs presented here for large phytoplankton.

By accounting for the acceleration of enzymatic reactions alongside photosystem damage as a function of temperature, our model captures observed growth and carbon fixation rates of various diatom species as well as the observed decrease in the carbon use efficiency with warming (Fig. 2, A to C). In agreement with empirical data, the model also predicts an increase in the chlorophyll content per unit of biovolume, a decline in the nitrogen-to-carbon ratio of the cell, and a decrease in cell size as temperature increases (Fig. 2, D to F). These responses are independent of the respiratory pathway used by the cell (Fig. 2 and fig. S1) and are also observed in small phytoplankton simulations (fig. S2). The model then allows us to tease apart the underlying molecular mechanisms driving these commonly reported relationships between traits and temperature.

### Shifts in investment with increasing temperatures

**Fig. 3.**
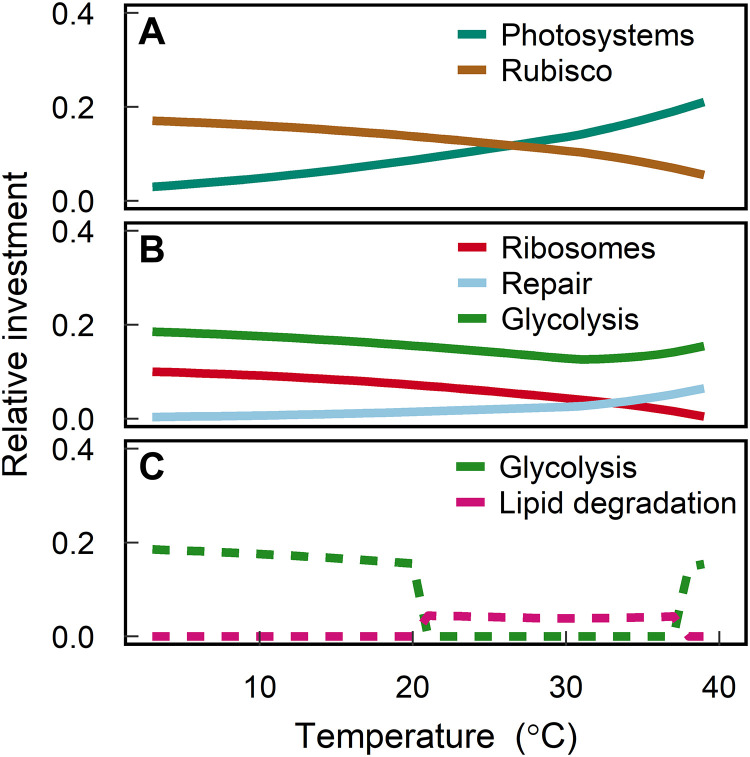
Optimal proteome investments. Changes in the relative proteome investment in different protein pools as a function of acclimated temperature. Solid lines (**A** and **B**) correspond to the investments when glycolysis is the only respiratory pathway available to the cell (investments are qualitatively the same when lipid degradation is the only respiratory pathway available; fig. S3). Metabolic switches are observed when the cell is given the option to choose between glycolysis or lipid degradation to fuel respiration (**C**). Model output is shown for the model calibrated to data from (*13*) with parameter values given in table S3.

Differential temperature dependencies of cellular processes play a critical role in observed trait relationships as the cell acclimates to warming. If temperature had the same effect on all cellular processes, no shift in allocation would occur, as increased energy generation would keep pace with increased respiration demands. This result would be inconsistent with observations (Table 1). In reality, temperature-independent photon absorption by photosystems is decoupled from the temperature-dependent enzyme kinetics of rubisco resulting in an energetic imbalance. Thus, as temperature increases, the optimal cell must invest more in light-harvesting proteins and divest resources away from rubisco to maximize growth rates (Fig. 3A and fig. S3A). The model is able to elucidate the mechanisms driving the observed shifts in rubisco gene transcripts and photosynthesis-antenna proteins under increased temperatures (Table 1) (*18*, *19*, *27*) as well as the observed increase in chlorophyll-a per biovolume as a function of temperature (Fig. 2D). Photosystem damage due to temperature stress exacerbates this energetic imbalance as the cell must also invest in more repair proteins as temperature increases (Fig. 3B, fig. S3B, and Table 1) (*18*, *19*, *27*) to avoid the excessive accumulation of irreversibly damaged photosystems (fig. S4). We explore the sensitivity of the model to the rate of damage and show that our main results do not change if photosystems and all other protein pools are assumed to be damaged at the same rate or if all pools are damaged but photosystems are damaged at a higher rate. These modified versions of the model incur a higher penalty in growth rates as temperatures increase due to the greater investment in repair proteins and respiration that is required (fig. S5).

The optimal cell compensates for the need to increase investment in photosystems and repair proteins as temperatures increase by divesting resources away from other parts of the proteome. Increased kinetic energy results in more efficient pathways for protein synthesis; thus, the optimal cell can decrease the relative investment in ribosomes without affecting growth rates (Fig. 3B and fig. S3B). Thus, the model predicts a decrease in the concentration of ribosomes with increasing temperatures (fig. S6), in agreement with empirical data (Table 1) (*19*, *27*, *28*) and previous modeling results (*28*). Our findings build on the modeling work by Toseland *et al.* (*28*) to show that the decrease in ribosomes is ultimately mediated by the need to invest more in photosystems and repair proteins with warming as a result of energetic imbalances. While this appears to be a general pattern, some studies observed an increase in total RNA content with increases in temperature (Table 1) (*13*, *29*), highlighting the need for further investigations into possible alternative metabolic strategies. The increased investment in photosystems and the resulting decreased investment in ribosomes contribute to the observed decrease in the nitrogen-to-carbon ratio of the cell with increasing temperatures (Fig. 2E), as photosystems are less nitrogen rich than ribosomes (table S2).

As temperature increases, the optimal cell initially divests resources away from respiratory pathways to accommodate the increased photosystem demand. This pattern is ultimately reversed as temperatures increase to near-fatal temperatures due to exponentially increasing metabolic demands (Fig. 3B and fig. S3B), which we discuss more below. If the cell is given a choice between fueling dark respiration with glycolysis or lipid degradation, the optimal cell switches from using glycolysis to using lipid degradation at a critical high temperature (Fig. 3C). This prediction is an emergent property of the model and provides mechanistic insight that is consistent with transcriptomic and proteomic evidence from cultured isolates (Table 1) in which glycolysis was found to be up-regulated in colder environments (*29*, *30*), while lipid degradation was up-regulated in warmer environments (*29*). Here, we suggest that this switch arises due to a trade-off between energy demands, protein requirements, and space.

To meet increasing energy demands due to rising temperatures, a cell must balance the relative investments in uptake and growth. While it costs less energy to build carbohydrates, lipids store more energy per mole of carbon (*31*) and also take up less space in the cell (*17*). The model predicts that lipids are preferentially used for respiration at higher temperatures to support higher metabolic demands while optimizing for smaller cell sizes (fig. S7), which helps to maintain a high surface area–to–volume ratio (see discussion below). By using lipid degradation instead of glycolysis, the cell can also increase its carbon use efficiency and the relative concentration of photosystems in the cell (fig. S7). The proteome model offers a unique framework to test the boundaries of this trade-off and the underlying mechanisms, which are otherwise hard to disentangle experimentally. Specifically, the model allows us to assess responses over a wide range of experimental conditions and parameter values and identify different growth regimes, whereas such experiments are challenging to conduct and are costly in terms of time and resources.

Once temperature increases to a near-fatal temperature, the optimal cell shifts into a third regime. In this regime, both respiration demand and stress due to photosystem damage are excessively high. Under these conditions, the optimal cell stores lipids to regulate stress rather than to fuel respiration and switches back to glycolysis to meet respiration demands (Fig. 3C). Glycolysis was found to be up-regulated at near-fatal temperatures in *Chlamydomonas reinhardtii* (Table 1) (*18*). The required carbohydrate storage to fuel dark respiration in this extreme stress case results in larger optimal cell sizes (fig. S7). A cell can avoid entering into this third regime by increasing the efficiency of the lipid degradation pathway (fig. S8). A shortcoming of the proteome allocation model is that it optimizes assuming a steady-state environment. As a result, the model never predicts the utilization of both the lipid degradation and glycolysis pathways, resulting in the metabolic switch (Fig. 3C and fig. S7). In reality, we expect that fluctuating environmental conditions would generate a smooth transition in which both pathways are used over the transition range.

### Changes in cell size due to warming

**Fig. 4.**
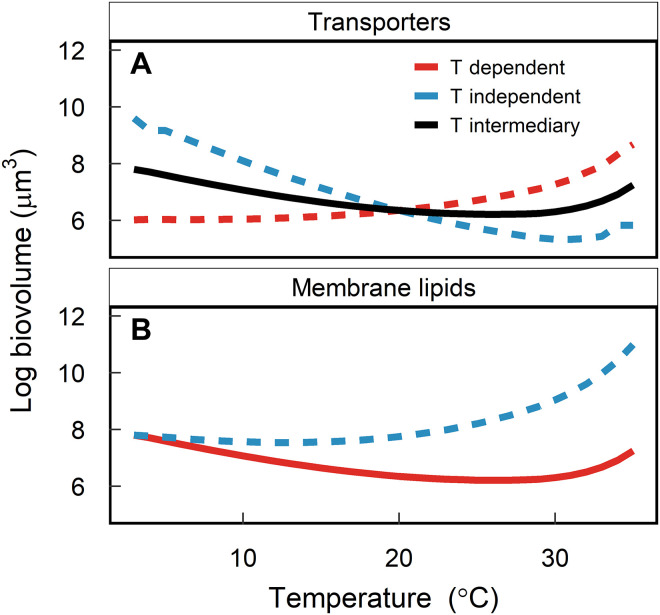
Cell size depends on the thermal dependencies of membrane components. Changes in cellular biovolume depend on the thermal dependencies of nitrogen transporters (**A**) and membrane lipids (**B**). Solid lines indicate the baseline run (shown in Figs. 2 and 3), while dashed lines indicate the sensitivity runs. In (A), the T-dependent run (red dashed line) uses the same temperature sensitivity for transporters as other reactions. To provide the other extreme case, runs in which transporters are assumed to be independent of temperature are shown (blue dashed line). The baseline uses an intermediary scenario for nutrient transporters (see Fig. 1B). (B) uses the intermediate temperature sensitivity for transporters [black line in (A)] and shows model simulations where lipid saturation does not change with temperature (blue dashed line) and where membrane lipids shift from unsaturated to saturated as temperatures increase (red line). Model output is shown for the model calibrated to data from (*13*) with parameter values given in table S3.

Our model provides insights into the mechanisms driving changes in cell size with increasing temperature. We find that the thermal dependency for nitrogen uptake relative to other reactions (Fig. 1B) plays an important role in the observed changes in cell size with warming (Fig. 2F). Specifically, if the thermal dependency of nutrient uptake is equal to that of other metabolic processes (e.g., ribosomes), as temperatures increase, the cell can maximize growth rate by divesting resources away from transporters to invest in the synthesis and repair of photosystems. This results in a decrease in the relative concentration of transporters per unit of biovolume as temperatures increase (fig. S9A) and, consequently, in a smaller surface area–to–volume ratio and larger optimal cell size (Fig. 4A). This is opposite to the observed trends of decreasing cell size with increasing temperatures even under replete nutrient conditions (*11*). On the other hand, if we assume that nitrogen transport into the cell is less sensitive to temperature than other metabolic processes, nitrogen uptake is less efficient than other pathways as temperatures increase and thus the cell must increase the relative concentration of transporters to maintain the same flux of nitrogen into the cell (fig. S9A). This results in a larger surface area–to–volume ratio and smaller optimal cell size (Fig. 4A). This hypothesis is supported by proteomic analysis that found nitrogen transporters to be up-regulated with warming even under replete nutrient conditions (Table 1) (*19*, *30*).

The proposed mechanism relating energetic imbalances, differential thermal dependencies, and cell size is also consistent with experimental evidence that the thermal dependency of nutrient uptake is lower than that of both carbon fixation and growth (*32*, *33*). Similarly, isotope incorporation studies and enzyme assays showed that the thermal dependency of nitrate uptake by the diatom *Thalassiosira pseudonana* matched the thermal dependency of nitrate reductase activity, which was lower than that of growth (*34*). Specifically, the *Q*_10_ (the factor by which metabolic rates increase by a 10°C rise in temperature) for nitrate reductase activity was found to be 1.8 (*35*), while the *Q*_10_ values for rubisco and respiration are approximately 2.4 and 3.0, respectively (*23*). Here, we halved the activation energy *E*_a_ (Eq. 4) of transporters relative to other processes, but the model results are qualitatively the same with different decreases in *E*_a_ (fig. S10). The model allows us to understand the trade-offs associated with this lower *Q*_10_ value and resulting impact on other traits.

We hypothesize that cell size is also affected by the lipid composition (saturated versus unsaturated) of the membrane. In the model, the surface area of the cell is calculated as a function of the concentration of membrane lipids and transporters. If we assume that there is no change in lipid saturation as temperature increases, then the cell maintains a constant ratio between transporters and lipids in the membrane across the temperature range. However, when we incorporate the need for cells to switch from unsaturated to saturated lipids for membrane stability (Table 1) (*18*, *19*, *27*), the relative concentration of lipids per unit area of membrane increases (fig. S9B). Saturated lipids take up less space compared to unsaturated lipids because they lack kinked hydrocarbon chains (*17*). Therefore, to maintain a constant spacing between transporters in the membrane, the ratio of lipids to transporters must increase with increasing temperature (Eq. 27). When this change to saturated lipids at high temperatures is included in the model (Eqs. 26 and 27), the surface area–to–volume ratio of the cell increases, resulting in smaller optimal cell sizes (Fig. 4B) to accommodate the same concentration of transporters and a higher concentration of lipids in the membrane.

The model predicts that cells get larger again at near-fatal temperatures across all modeled scenarios (Figs. 2F and 4). This occurs during the third regime described above. Energetic constraints at high temperatures force the cell to heavily invest in respiration and carbon storage, decreasing the concentration of transporters and membrane lipids per unit of biovolume (fig. S9). Consequently, the cell decreases its surface area–to–volume ratio to accommodate the required carbon storage and energy investment.

### Drivers of thermal adaptation

Cells respond to shifts in environmental conditions first by acclimating (rearrangement of metabolism through phenotypic changes) and then by adapting (genetic changes). The proteome model allows us to study both responses. The acclimation response is captured by the different allocation strategies predicted at different temperatures (i.e., those described above) based on a set of constant parameter values (table S2). However, by altering underlying parameters (table S3), we can also use the model to understand the adaptive responses of phytoplankton. For example, understanding which model parameters can be altered to capture observed differences between phytoplankton species (Fig. 2 and fig. S2) can provide insight into different adaptive strategies. This is done systematically to assess the sensitivity of the model results to all free parameters (figs. S10 to S16). To investigate ways in which a single phytoplankton species adapts to high temperatures, we conduct an additional set of model simulations. Here, we allow changes (akin to mutations) in parameters (akin to genes) associated with the (i) activation energy *E*_a_, (ii) maximum total lipid content *ctag*_max_, and (iii) maximum turnover rates *k*_ref_ (table S3). We compare our results against data from Schaum *et al.* (*13*) in which diatom strains of *T. pseudonana* were grown for 300 generations at the following temperatures: 22°C (control), 26°C (moderate warming), and 32°C (severe warming). This analysis allows us to uncover the mechanistic links between cellular adaptive strategies and trade-offs.

**Fig. 5.**
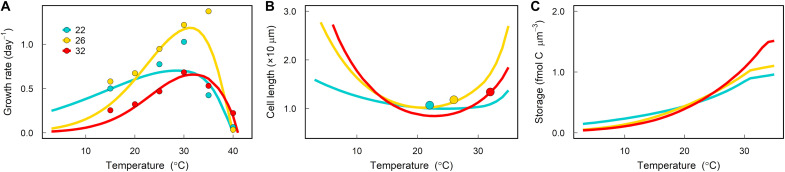
Thermal acclimation of phytoplankton evolved at different temperatures. Model output (lines) compared against data from an experimental evolution study (symbols) in which the diatom *T. pseudonana* were grown for 300 generations at the following temperatures: 22°C (control), 26°C (moderate warming), and 32°C (severe warming) (*13*). (**A**) Growth rate, (**B**) cell length, and (**C**) carbon storage, which includes both carbohydrates and lipids required to meet dark respiration costs and stored lipids for mitigating oxidative stress.

Empirical evolution studies have shown that phytoplankton can adapt to high temperatures by increasing their optimum growth temperature *T*_opt_ (*10*, *13*, *36*, *37*). The capacity of enzymes to evolve thermostability is well known, especially in the field of protein engineering (*38*). This response can be captured in the model by increasing the activation energy *E*_a_, which sets the slope at which metabolic rates increase exponentially with increasing temperature (table S3). This adaptation allows higher growth rates at high temperatures but comes at the cost of reduced growth rates at lower temperatures due to slower kinetics (Fig. 5A). In addition to this known trade-off, the model proposes additional trade-offs associated with evolving enzymes that perform better at high temperatures. Because the thermal dependency of nutrient transport into the cell is lower than other reactions in the model (see discussion above), warm-adapted cells can minimize the impact of this trade-off by devoting more resources to rubisco and ribosomes and divesting resources away from transporters at cooler temperatures. However, this requires the warm-adapted cells to be larger than their ancestors (Fig. 5B). Furthermore, although cells can evolve enzymes that are more stable at higher temperatures, metabolic rates still increase exponentially with temperature in the warm-adapted cells, resulting in high respiration demands. The optimal warm-adapted cell must then store even more carbon than the ancestor cell to fuel respiration as temperatures increase (Fig. 5C). Thus, the model provides a mechanistic explanation for why, at high temperature, the warm-adapted cells both grow faster and are larger than the ancestral strains (Fig. 5B, blue versus yellow).

The model identifies an additional trade-off associated with warm temperature adaptation. To adapt to critically high temperatures (i.e., grow faster and increase carbon use efficiency), cells must also invest in mechanisms to minimize and repair damage resulting from oxidative and heat stress (*13*), thus divesting resources away from other intracellular processes. While there are many ways in which a cell can invest resources to mitigate heat and oxidative stress, here, we test two strategies: (i) the use of lipids to sequester ROS and (ii) the use of antioxidant enzymes to decrease oxidative stress. Both strategies result in qualitatively similar results. Here, we present the lipid strategy based on results from two experimental evolution studies that found increased lipid content in warm-adapted cells (*15*, *16*). The results from the antioxidant enzyme simulations are provided and discussed in the Supplementary Materials (fig. S12; see the “Antioxidant enzyme simulation” section in Supplementary Text).

The model is able to capture the trade-offs associated with adaptation to both moderate (26°C) and extreme (32°C) changes in temperature. Increasing just the maximum lipid content (*ctag*_max_) decreases the cost of heat-damaged photosystems *e*_d_ (table S3), allowing the 32°C evolved cells to sustain growth at the previously fatal temperature of 40°C (fig. S11A). This growth at extreme temperatures comes at the cost of reduced growth across the growth curve relative to the 26°C evolved model (e.g., maximum growth of 1.02 day^−1^ compared to 1.14 day^−1^ for the 26°C evolved model). It also requires increased cell sizes to accommodate higher lipid storage (fig. S11B). By shifting the thermal dependency of the reactions (higher *E*_a_) and evolving higher lipid content, the cell can obtain higher growth rates relative to the simulations where only maximum lipid content increased, albeit still slightly lower than the 26°C evolved model (fig. S11A). The model dynamics are consistent with the observed physiological shifts in warm-adapted cells (*13*) and suggests a mechanism for the observed changes. Specifically, to minimize damage at high temperatures and support high respiration demands, the cell must invest in the synthesis of stored lipids, which take up cellular space and divest carbon away from growth (fig. S11C).

The model provides an additional prediction. Because of the high cost of respiration under severe warming (32°C), it is advantageous to slow all reaction rates to keep energy demands lower at high temperatures, decrease respiration rates, and, thus, require less carbon storage, which allows the cell to maintain a smaller size (fig. S11B). Specifically, the model is only able to capture the larger decrease in growth rates across the entire growth curve observed in the experimental data (red line in Fig. 5A) by also decreasing the maximum turnover rates of all reactions (i.e., *k*_ref_; table S3). Decreases in *k*_ref_ for high temperature–adapted strains is consistent with previous findings (*13*, *36*, *10*). Experimental evolution studies for a variety of different phytoplankton species [*Chlorella vulgaris* (*36*), *Ostreococcus tauri* (*10*), *Synechococcus* (*10*), and *T. pseudonana* (*13*)] all show that photosynthesis and respiration rates decreased in high temperature–adapted strains. While an increase in carbon use efficiency for high temperature–adapted strains relative to ancestral strains appears to be a robust response, the cellular trade-offs behind this response were not previously known. More work is needed to understand additional trade-offs associated with adaptations to these extreme temperatures, but the model provides a useful tool for teasing mechanisms apart.

## DISCUSSION

**Fig. 6.**
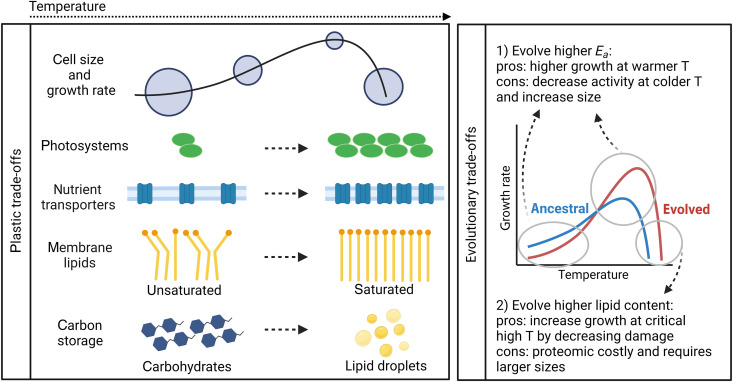
Phytoplankton thermal trade-offs. Phytoplankton respond plastically to increases in temperature by decreasing cell size; by increasing photosystems, transporters, and the saturation of membrane lipids; and by storing lipids instead of carbohydrates. At critically high temperatures, the cell gets larger again because it must maximize investment in carbon storage to fuel high respiration rates. To adapt to warming, phytoplankton can evolve higher *E*_a_ so that enzymes function better at high temperatures at the cost of higher carbon storage and lower growth at colder temperatures. Cells can also evolve higher lipid content or use other metabolisms such as antioxidant enzymes to decrease cellular damage, which requires larger sizes. Schematic created with BioRender.com.

Our proteome model provides insight into the underlying cellular mechanisms behind observed plastic and evolved phytoplankton trait changes to shifts in temperature (Fig. 6). Identifying which trade-offs are hard constraints determined by energetic or biophysical limitations versus malleable constraints that can be overcome via evolution generates hypotheses for how phytoplankton might adapt to a warm ocean and the resulting impact on ecosystem processes (*13*, *37*). We make three important predictions. First, lipids are preferentially used for respiration as temperature increases because they have a higher recoverable energy and take up less space in the cell. This has potential implications for food web dynamics by producing high-energy prey. Second, changes in the saturation of membrane lipids and the lower thermal dependency of nutrient uptake explain why cells acclimate to warming by decreasing size. These trade-offs result from fundamental imbalances in cellular energetics and space. Third, phytoplankton might break the observed temperature-size correlation and evolve larger sizes under temperature stress by evolving metabolisms to mitigate intracellular stress at high temperatures at a cost to growth at lower temperatures. We hypothesize that increasing cell size may be an important strategy to allow for increased lipid content and minimize oxidative stress when faced with critically high temperatures.

### Why do phytoplankton acclimate to warming by decreasing cell size?

As predicted by theory and empirical evidence, we find that phytoplankton acclimate to warming by decreasing cell size (Fig. 6). Decreases in size as a function of temperature have been reported for diverse organisms, both across and within species; this response is so ubiquitous that it has been considered a universal ecological response to global warming (*3*), although this is debated (*39*). Decreases in the size of marine plankton due to warming have been explained by the accompanied effects on decreased nutrient availability due to increased stratification of the water column (*4*, *40*). However, decreases in cell size are also observed experimentally when warming is decoupled from nutrient limitation (*11*). Our model reveals that this is because, even under replete nutrient conditions, phytoplankton can become limited by nutrients due to the lower thermal dependency of nutrient uptake relative to other metabolic processes (*23*, *32*, *34*). Thus, as temperatures warm, a cell must increase the relative investment in nutrient transporters, as supported by previous proteomic analyses (*19*, *30*), to keep pace with increased metabolic demands. In addition, at higher temperatures, cells switch from unsaturated to saturated lipids (*24*), requiring a higher relative investment in the synthesis of saturated lipids (*18*, *19*, *27*) to maintain membrane integrity, which also decreases the optimal cell size.

We identify a trade-off between allocating cellular space to photosystems and carbon storage (increasing cellular volume) versus membrane components (increasing cellular surface area) as temperatures increase (Fig. 6). To manage this trade-off, we find that the cell switches from glycolysis to lipid degradation due to warming, as supported by empirical evidence (*29*, *30*). By choosing to store lipids instead of carbohydrates (*17*), phytoplankton fine-tune respiration (*41*) by more efficiently storing energy per mole of carbon as well as per unit of biovolume. This increases the cells’ carbon use efficiency and allows for smaller cell sizes at warmer temperatures. However, the model also predicts that cells should get larger again at critically high temperatures (Fig. 6). This has been observed empirically (*29*, *42*) and is usually attributed to cell cycle arrest just before cell death (*18*). Here, we propose that there is an additional mechanism at play. Cells must invest more in repair and respiration and thus carbon storage, divesting resources away from membrane components and investing resources in components that require larger cellular volume. Because lipids can help mitigate damage due to oxidative stress, our model predicts that the optimal cell switches back to glycolysis to fuel respiration, which promotes further increases in cell size at extremely high temperatures (fig. S7). While cells typically switch from glycolysis to lipid degradation as temperatures warm, a previous study found that phytoplankton up-regulated glycolysis under extreme heat stress (*18*).

The respiratory pathways used by phytoplankton might also affect the oxygen cycling. Because of the lower oxygen content of lipids relative to carbohydrates (*43*), the molar ratio of oxygen to organic carbon consumed during respiration is higher for lipids. This can have important consequences to the oxygen cycling in the global ocean, decreasing the concentration of dissolved oxygen and increasing low-oxygen zones due to warming, particularly in the oligotrophic gyres (*44*).

### Can phytoplankton adapt to warming without shrinking?

One might ask: Is the observed relationship between small size and warmer temperatures strongly constrained, or is there a way for phytoplankton to adapt to warming without shrinking? Schaum *et al.* (*13*) found that while high temperature–acclimated cells decreased cell size, high temperature–adapted populations evolved larger cell sizes and higher growth rates at elevated temperatures compared to the ancestral population. Using the proteome allocation model, we reveal the mechanisms and trade-offs both behind the observed trend of decreases in cell size with temperature and how phytoplankton might alternatively be able to get larger under temperature stress, thus challenging the temperature size rule. We found that to evolve higher growth rates at warmer temperatures, phytoplankton must (i) store more carbon to fuel higher rates of respiration and/or (ii) decrease heat damage by evolving antioxidant responses such as higher lipid content or increased production of antioxidant enzymes (Fig. 6). Both strategies require allocating more cellular space toward storage, which requires larger cell sizes.

The predictions described above have important consequences to ecosystem functioning. Large phytoplankton with high lipid content can have a positive impact on the higher trophic levels in the marine food web. While larger phytoplankton can help support larger predators, higher lipid content can translate into higher food quality. If cells can adapt in this way, we would expect higher trophic transfer efficiency and larger export due to the overall high contribution of larger particles to the biological pump, contrary to short-term predictions (*15*, *16*). However, under the multistressor environment of high temperature and nutrient limitation, previous empirical work has found that phytoplankton thermal responses are more strongly constrained (*45*) and that thermal adaptation was not possible (*46*). These previous studies typically assume a constant environment. The question then becomes if large cell sizes could arise as an alternative adaptive strategy in environments with frequent and high variations in temperature and pulses of nutrients. In such an environment, larger cells would be able to store more carbon to regulate oxidative stress to support higher growth during warmer periods. Even the most oligotrophic seas are influenced by nutrient pulses derived from meso- and submesoscale features (*47*). Thus, large phytoplankton could benefit from storing nutrients during these episodes, suggesting that there might be a niche for being large in a future warmer ocean.

### Future directions

Our findings are grounded in a large body of theory (*2*, *48*) and lessons from trait ecology (*11*, *49*) as well as based on insights from experimental evolution studies (*13*, *15*) and ‘omics approaches (*24*, *29*, *41*). By integrating these fields, we investigate the gaps in knowledge related to the molecular trade-offs that constrain both short-term acclimation and evolutionary adaptation of phytoplankton due to warming. Previous work has shown that proteome models can provide insight into microbial choices across different environments (*26*, *50*–*54*). Here, we show how the proteome allocation model can be used to investigate short-term (acclimated) and long-term (adaptive) responses and provide mechanistic hypotheses related to underlying trade-offs. Future studies designed to specifically test these hypotheses against proteomic data will important. Specifically, we need more studies that quantify proteomic and metabolic responses across multiple temperatures to accurately assess the underlying mechanisms. We hypothesize that the growth strategy of cells shift multiple times as temperatures increase (here, we discuss three regimes) such that experiments comparing only two temperatures can be difficult to interpret and contrast with other studies.

This model provides a powerful tool for testing metabolic strategies and understanding the conditions when one strategy is favorable over another. Here, we contrast strategies for fueling dark respiration and two potential antioxidant strategies. However, the model can easily be expanded to explicitly test trade-offs associated with other metabolic strategies within the context of the general space and energy constraints discussed here. For example, the model could provide insight into the ability to combine photosynthesis with the consumption of prey (mixotrophy) and how these diverse metabolic strategies can drive different thermal responses among protists. This modeling framework could also be used to investigate within species trade-offs across different environments and across species trade-offs driving differences in functional group traits. A shortcoming of this modeling approach is that the model only predicts the steady-state optimal solution. An exciting direction for future research is to modify the model to consider strategies that prepare for future environments (e.g., luxury nutrient uptake) or fluctuating conditions. Ultimately, proteome models can inform the key trait trade-offs that should be included in large-scale ecosystem models (*28*, *55*), allowing us to better understand how fine-scale processes scale up to influence global biogeochemical cycles. This is particularly critical because both trait values and the correlation between traits will constrain the possible evolutionary outcomes of phytoplankton in a warmer ocean (*56*).

## MATERIALS AND METHODS

### The proteome allocation problem

Our coarse-grained model is built as a constrained optimization problem that maximizes the steady-state growth rate of a generic photosynthetic cell considering temperature, irradiance levels, and external dissolved inorganic nitrogen and carbon concentrations. While the model does not dynamically simulate light-dark cycles, it requires the cell to prepare for the dark part of the light cycle, i.e., the cell must produce and store enough chemical energy during the light period to account for dark respiration. The model is based on previous proteome models developed for heterotrophic and phototrophic metabolisms (*26*, *50*–*54*). For each environmental condition, the model optimizes the following state variables: the intracellular concentration of proteins (*p*) and other macromolecules (*c*), the relative investments in the different proteins (ϕ), the volume–to–surface area ratio of the cell (β), and the fractions of the lipid synthesis flux designated to build membrane lipids (α_lm_) and stored lipids (α_tag_) and to fuel the lipid degradation pathway (α_ld_) (table S1). Our work extends previous models to account for temperature effects, lipid and energy metabolisms, and space constraints in the model, allowing us to predict changes in phytoplankton cell size and stoichiometry as a function of temperature. Parameter values are presented in table S2 for those that were kept constant throughout our simulations and in table S3 for those that were fitted to observational data. More details on the choice of parameter values are given in Supplementary Methods (tables S6 to S14).

### The mathematical model

The model assumes a cell that is growing exponentially at the highest growth rate possible given a specific constant environmental setting. Thus, for each macromolecule in the cell (*c*), the sum of the rates (*v*) of all synthesis processes minus the sum of the rates of all degradation processes is balanced by the cellular growth rate μ

$$\frac{dc}{dt}=\sum v_{\mathrm{synthesis}}-\sum v_{\mathrm{degradation}}-\mu c=0$$ (1)

We solve for each cellular pool concentration (molecules per cubic micrometer of biovolume) that maximizes μ. The reaction rates catalyzed by a given protein *i* are represented by a Michaelis-Menten–type equation

$$v_{i}=k_{i}p_{i}\frac{S_{i}}{S_{i}+K_{i}}$$ (2)

in which *k_i_* is the maximum temperature corrected turnover rate per protein. *S_i_* is the substrate concentration, *K_i_* is the half-saturation constant, and *p_i_* is the concentration of protein *i* in molecules of protein *i* per cubic micrometer of biovolume. The glycolysis and lipid degradation fluxes estimated to meet dark respiration costs are an exception because all glucose and lipid storage will be used to fuel respiration during the night and thus Eq. 2 can be simplified as *v_i_* = *k_i_p_i_* for these pools.

We assume that all enzymatic rates in the cell increase with temperature following the Arrhenius equation

$$k_{i}=k_{{\mathrm{ref}}_{i}}\gamma_{Ta}$$ (3)

$$\gamma_{Ta}=\exp\left[\frac{E_{a}}{R}\left(\frac{1}{T_{\mathrm{ref}}}-\frac{1}{T}\right)\right]$$ (4)

in which *k*_ref*_i_*_ is the maximum turnover rate at a reference temperature *T*_ref_ (in kelvin), *E*_a_ is the activation energy (eV), *R* is Boltzmann’s constant (eV K^−1^), and *T* is the actual temperature (K). The only exception is the maximum rate of photosynthesis (*k*_p_), which we assume is independent of temperature (*23*). Simulations were run where *E*_a_ was constant for all reactions (except photosynthesis) and where *E*_a_ varied for different enzymes (e.g., Fig. 1B).

All protein pools *p_i_* are synthesized by ribosomes (*p*_ri_), including ribosomes themselves. The total protein synthesis flux catalyzed by ribosomes *v*_ri_ (molecules of amino acids μm^−3^ min^−1^) is partitioned among the different proteins according to the relative proteome investments ϕ*_i_*. Because our model does not simulate the full proteome, we assume that a fraction of the proteome devoted to other protein pools (*p*_other_; table S2) is constant, and thus, the sum of all proteome relative investments is equal to (1 − ϕ_other_)

$$\sum \phi_{i}=(1-\phi_{\mathrm{other}})=0.5$$ (5)

Our results are not sensitive to the choice of *p*_other_ (fig. S13). One can rewrite Eq. 1 for a given protein pool *i* as the following

$$\phi_{i}v_{\mathrm{ri}}\frac{1}{\eta_{i}}-\mu p_{i}=0$$ (6)

in which η*_i_* is the molecular weight of a given protein (number of amino acids per protein). The model is fully defined in the Supplementary Materials, and a detailed description of the different metabolic pathways is given in the following sections.

### Protein synthesis

The protein synthesis flux (*v*_ri_; molecules of amino acids μm^−3^ min^−1^) is catalyzed by ribosomes and requires both internal carbon (*c*_ic_) and internal nitrogen (*c*_in_)

$$v_{\mathrm{ri}}=k_{\mathrm{ri}}p_{\mathrm{ri}}\frac{c_{\mathrm{ic}}}{c_{\mathrm{ic}}+K_{\mathrm{ic}}}\frac{c_{\mathrm{in}}}{c_{\mathrm{in}}+K_{\mathrm{in}}}$$ (7)

The cellular nitrogen to carbon demand ($\frac{\eta_{\mathrm{aan}}}{\eta_{\mathrm{aac}}}$) is defined as

$$\frac{\eta_{\mathrm{aan}}}{\eta_{\mathrm{aac}}}=\sum \phi_{i}q_{i}$$ (8)

in which the subscript *i* represents protein pool *i*, and *q* is the ratio of nitrogen to carbon for that protein pool. We use an average value for the carbon content of the protein pools (η_aac_, molecules of C per amino acid; table S2) and solve for η_aan_ (molecules of N per amino acid). The above equation thus controls the consumption rate of carbon and nitrogen used in the synthesis of proteins to meet the optimal stoichiometry of the cell.

### Carbon metabolism

The conversion of dissolved inorganic carbon (DIC) into a generic pool of organic carbon (*c*_ic_) is mediated by the rubisco protein pool (*p*_ru_) such that the flux of carbon fixation *v*_ru_ (molecules of C μm^−3^ min^−1^) is given by

$$v_{\mathrm{ru}}=k_{\mathrm{ru}}p_{\mathrm{ru}}\frac{\mathrm{DIC}}{\mathrm{DIC}+K_{\mathrm{ru}}}$$ (9)

For the model results presented here, we assume that carbon is not limited: DIC >> *K*_ru_, so *v*_ru_ ≈ *k*_ru_*p*_ru_. This internal organic carbon (*c*_ic_) is used in three different reactions: (i) protein synthesis (*v*_ri_) catalyzed by ribosomes, (ii) lipid synthesis (*v*_lb_) catalyzed by the protein pool *p*_lb_, and (iii) glycolysis (*v*_gl_) catalyzed by the protein pool *p*_gl_ to fuel cellular respiration during the night. The reaction rates *v*_lb_ and *v*_gl_ are in units of molecules of C μm^−3^ min^−1^.

$$v_{\mathrm{lb}}=k_{\mathrm{lb}}p_{\mathrm{lb}}\frac{c_{\mathrm{ic}}}{c_{\mathrm{ic}}+K_{\mathrm{lb}}}$$ (10)

$$v_{\mathrm{gl}}=k_{\mathrm{gl}}p_{\mathrm{gl}}$$ (11)

The glycolysis flux can be simplified as *v*_gl_ = *k*_gl_*p*_gl_ because we assume that all glucose will be used to fuel respiration during the night.

We can then write the equation for the internal pool of carbon

$$\frac{{dc}_{\mathrm{ic}}}{dt}=v_{\mathrm{ru}}-v_{\mathrm{ri}}\eta_{\mathrm{aac}}-v_{\mathrm{lb}}-v_{\mathrm{gl}}-\mu c_{\mathrm{ic}}=0$$ (12)

in which *v*_ri_ is multiplied by η_aac_ to convert the amino acid flux to carbon units.

### Nitrogen metabolism

Dissolved inorganic nitrogen (DIN) is imported into the cell (*v*_tr_; molecules of N μm^−3^ min^−1^) by nitrogen transporters (*p*_tr_) localized in the membrane

$$v_{\mathrm{tr}}=k_{\mathrm{tr}}p_{\mathrm{tr}}\frac{\mathrm{DIN}}{\mathrm{DIN}+K_{\mathrm{tr}}}$$ (13)

$$v_{\mathrm{tr}}\approx k_{\mathrm{tr}}p_{\mathrm{tr}}$$ (14)

As we focus here on the impact of temperature on cell metabolism, we assume nitrogen is not limiting so that DIN >> *K*_tr_ and *v*_tr_ ≈ *k*_tr_*p*_tr_. Internal nitrogen (*c*_in_) is subsequently consumed by the protein synthesis pathway (*v*_ri_) such that the internal pool of nitrogen can be obtained from

$$\frac{{dc}_{\mathrm{in}}}{dt}=v_{\mathrm{tr}}-v_{\mathrm{ri}}\eta_{\mathrm{aan}}-\mu c_{\mathrm{in}}=0$$ (15)

in which *v*_ri_ is multiplied by η_aan_ to convert the amino acid flux to nitrogen units.

### Cell stoichiometry

By simulating carbon and nitrogen metabolisms and varying the nitrogen content per amino acid (η_aan_) according to the nitrogen to carbon demand, it is possible to estimate the overall N:C quota of the photosynthetic cell (*q*_cell_) by computing the weighted average

$$q_{\mathrm{cell}}=\frac{\sum p_{i}\eta_{i}q_{i}\eta_{\mathrm{aac}}+c_{\mathrm{in}}}{\sum p_{i}\eta_{i}\eta_{\mathrm{aac}}+c_{\mathrm{ic}}+(c_{\mathrm{lm}}+c_{\mathrm{li}}+c_{\mathrm{tag}})\eta_{\mathrm{lic}}+c_{\mathrm{gu}}\eta_{\mathrm{guc}}}$$ (16)

in which *i* represents protein pools, *p* is protein concentration, η is the protein molecular weight in units of amino acids per protein, and *q* is the protein nitrogen-to-carbon ratio (see table S2). It is important to note that *q*_cell_ represents the light proteome, thus accounting for the carbon storage (*c*_gu_ + *c*_li_) that is needed to meet dark respiration. As shown by previous studies, carbon storage required for dark metabolism can account for a large amount of carbon in the cell (*17*, *51*).

### Photosynthesis

Photosystems (*p*_p_) absorb photons at the rate of *v_p_* (molecules of photon μm^−3^ min^−1^) converting light (*I*) energy into chemical energy at the rate of *v*_ep_ [molecules of adenosine 5′-triphosphate (ATP) μm^−3^ min^−1^], as follows

$$v_{p}=k_{p}p_{p}\frac{I}{I+K_{p}}$$ (17)

$$v_{\mathrm{ep}}=v_{p}e_{p}$$ (18)

in which *e*_p_ is the amount of chemical energy produced per photon absorbed.

Photosystems are assumed to be damaged by heat. Our model captures several aspects of photosystem damage: reversible photosystem damage, irreversible photosystem damage, and minimization of damage by reducing oxidative stress (*25*). It is known that photosystems are susceptible to heat damage, more specifically the D1 protein within photosystem II. As heat stress increases, photosystem damage increases. We represent this process in the model as a temperature-dependent damage term after (*26*)

$$k_{d}=k_{{\mathrm{ref}}_{d}}\gamma_{Td}$$ (19)

$$\gamma_{Td}=\exp\left[\frac{E_{d}}{R}\left(\frac{1}{T_{d}}-\frac{1}{T}\right)\right]$$ (20)

in which *k*_d_ is the maximum rate of damage at a given temperature, *k*_ref_d__ is the maximum rate of damage at a reference temperature *T*_ref_, and *E*_d_ is the deactivation energy that sets the slope at which temperature increases the damage of photosystems above *T*_d_.

We allow the cell to mitigate photosystem damage caused by heat stress either through stored lipids (*c*_tag_) or by producing antioxidant enzymes (*p*_ox_). Here, we describe the model version that uses lipids to mitigate oxidative stress. The alternative model formulation is described in Supplementary Text (see the “Antioxidant enzyme simulations” section). Lipid droplets act as a sink for ROS and have been observed to play a role in the repair of damaged photosystems (*18*). The cell can thus choose to maximize the concentration of stored lipids up to *c*_tag_max__ in the cell to minimize the rate of damage of photosystems as temperature changes, or it can choose to store less lipids at the cost of higher damage (Eqs. 21 and 22). Thus, the actual rate of damage *v*_d_ depends on *k*_d_ (Eq. 19), on the concentration of photosystems *p*_p_, and on the concentration of stored lipids *c*_tag_

$$c_{\mathrm{tag}}\leq c_{{\mathrm{tag}}_{\min}}+c_{{\mathrm{tag}}_{\max}}$$ (21)

$$v_{d}=k_{d}p_{p}\frac{c_{{\mathrm{tag}}_{\max}}}{c_{\mathrm{tag}}}$$ (22)

in which the minimum concentration of stored lipids *c*_tag_min__ is needed to avoid *v*_d_ going to zero.

We assume that some damaged photosystems (*p*_dp_) can be repaired via repair proteins (*p*_re_). When heat stress is moderate, the photosystem can be repaired by removing and replacing the D1 protein using specific proteases (*25*); however, when heat stress is extreme, irreversible damage occurs. Both mechanisms are captured by our model and *p*_dp_ accounts for both the reversible and irreversibly damaged photosystems. The rate of repair (*v*_re_) is calculated as

$$v_{\mathrm{re}}=k_{\mathrm{re}}p_{\mathrm{re}}\frac{p_{\mathrm{dp}}}{p_{\mathrm{dp}}+p_{p}}$$ (23)

Considering that the repair processes are relatively fast compared to protein synthesis (*57*), the damage-repair processes can be assumed to converge to a quasi-steady state (*v*_re_ = *v_d_*), following Mairet *et al.* (*26*). This allows us to solve for the fraction of damaged photosystems

$$\frac{p_{dp}}{p_{dp}+p_{p}}=\frac{k_{d}p_{p}\gamma_{\mathrm{tag}}}{k_{\mathrm{re}}p_{\mathrm{re}}}$$ (24)

Last, the photosystems equation can be written as

$$\frac{d(p_{p}+p_{\mathrm{dp}})}{dt}=\phi_{p}v_{\mathrm{ri}}\frac{1}{\eta_{p}}-\mu (p_{p}+p_{\mathrm{dp}})=0$$ (25)

### Lipid metabolism

Lipids play three different roles in our model. First, we assume that the cell must invest in lipids to maintain membrane integrity following Molenaar *et al.* (*50*). This is implemented by assuming that the ratio between transporter proteins (*p*_tr_) and lipids (*c*_lm_) in the membrane must be equal to or lower than a certain value *M*_min_ + *M*

$$\frac{p_{\mathrm{tr}}}{c_{\mathrm{lm}}}\leq M_{\min}+M$$ (26)

*M*_min_ is the minimum ratio between *p*_tr_ and *c*_lm_. The *M* term captures the temperature dependence of lipid saturation. Specifically, the saturation state of lipids increases as a function of temperature (*18*, *21*). While unsaturated fatty acids have kinks in their molecular structure due to single or multiple double bonds, saturated fatty acids lack these kinks and thus can be more tightly packed together, occupying less space in the membrane. Because the constraint given in Eq. 26 controls the minimum spacing between transporters needed for membrane integrity, as temperatures increase, more lipids per surface area of membrane is needed. For simplicity, we assume that *M* decreases linearly as a function of temperature (*24*)

$$M=(M_{\max}-M_{\min})\left(\frac{T_{\max}-T}{T_{\max}-T_{\min}}\right)$$ (27)

in which *M*_max_ is the maximum transporter-to-lipid ratio in the membrane and *T*_max_ and *T*_min_ are the maximum and minimum temperatures, respectively, at which we perform our modeling experiments.

Second, we assume that stored lipids *c*_tag_ can be used to mitigate oxidative stress as described above (Eqs. 21 and 22). Third, lipids can fuel dark respiration through the lipid degradation pathway. We thus optimize for the fractions of the lipid synthesis flux (*v*_lb_) that are used to synthesize (i) membrane lipids (α_lm_), (ii) stored lipids that can mitigate damage (α_tag_), and (iii) lipids for dark respiration (α_ld_) so that

$$\alpha_{\mathrm{lm}}+\alpha_{\mathrm{tag}}+\alpha_{\mathrm{ld}}=1$$ (28)

We assume that the lipid degradation pathway must be catalyzed by the protein pool *p*_ld_ so that *v*_ld_ = *k*_ld_*p*_ld_ = α_ld_*v*_lb_. Last, we can define the equations for the membrane lipid and stored lipids pool as follows

$$\frac{{dc}_{\mathrm{lm}}}{dt}=\alpha_{\mathrm{lm}}v_{\mathrm{lb}}\frac{1}{\eta_{\mathrm{lic}}}-\mu c_{\mathrm{lm}}$$ (29)

$$\frac{{dc}_{\mathrm{tag}}}{dt}=\alpha_{\mathrm{tag}}v_{\mathrm{lb}}\frac{1}{\eta_{\mathrm{lic}}}-\mu c_{\mathrm{tag}}$$ (30)

in which *v*_lb_ (molecules of carbon μm^−3^ min^−1^) is multiplied by the inverse of η_lic_ (molecules of carbon per molecule of stored lipid) to convert the flux from carbon to lipid units.

### Energy metabolism

The energy metabolism of the cell is constrained based on reproduction and consumption terms for all cellular processes so that for each reaction rate *v_i_*, there is an associated energy conversion factor *e_i_* (table S2). The cell can obtain energy through three pathways: photochemistry, glycolysis, and lipid degradation. All other metabolic functions have an associated energetic cost. Energy fluxes are described in units of molecules of ATP μm^−3^ min ^−1^.

We first assume that light energy converted by photosystems (*e*_p_; ATP per photon) must meet the energy cost associated with rubisco activity (*e*_ru_; ATP per carbon)

$$e_{p}v_{p}\geq e_{\mathrm{ru}}v_{\mathrm{ru}}$$ (31)

After meeting rubisco costs, photosystems can also contribute to meet the energetic demands of other metabolic functions (*e*_cost_)

$$e_{\mathrm{cost}}\leq (e_{p}v_{p}-e_{\mathrm{ru}}v_{\mathrm{ru}})$$ (32)

in which the total energetic cost of the cell (*e*_cost_), excluding rubisco costs, can be defined as the sum of the metabolic fluxes *v_j_* multiplied by its respective energy costs *e_j_*

$$e_{\mathrm{cost}}=\sum e_{j}v_{j}$$ (33)

We assume that a fraction *f*_dr_ of *e*_cost_ happens in the dark and thus must be paid through either glycolysis (*v*_gl_) or lipid degradation (*v*_ld_)

$$e_{\mathrm{cost}}f_{\mathrm{dr}}\leq e_{\mathrm{gl}}v_{\mathrm{gl}}+e_{\mathrm{ld}}v_{\mathrm{ld}}$$ (34)

*f*_dr_ is a constant in our model and we assume it to be 25% of *e*_cost_. The amount of dark respiration relative to respiration occurring during the day is hard to constrain and varies by phytoplankton species and light-dark cycle. Falkowski and Owens (*58*) estimated dark respiration for six species of phytoplankton and found that it accounted on average for approximately 25% of gross photosynthesis, with a maximum of 50% being observed. Because this parameter is poorly constrained, we performed a sensitivity test to confirm that our choice for the fraction of respiration occurring in the dark does not alter our results (fig. S14). We find that as *f*_dr_ increases, the cell must increase the investment in respiration, decrease growth rates, and increase cell sizes to accommodate for carbon storage that fuels respiration.

Last, we can calculate the concentration of the storage pools for both glucose (*c*_gu_) and lipids (*c*_li_) needed to fuel respiration during the dark period

$$c_{\mathrm{gu}}=v_{\mathrm{gl}}\frac{1}{n_{\mathrm{guc}}}t_{d}$$ (35)

$$c_{\mathrm{li}}=v_{\mathrm{ld}}\frac{1}{n_{\mathrm{li}c}}t_{d}$$ (36)

in which *n*_guc_ is the number of molecules of carbon per molecule of glucose, *n*_lic_ is the number of molecules of carbon per molecule of stored lipid, and *t*_d_ is the duration in minutes of the dark period that the cell experiences over a day. Here, we assumed a 12-hour light/12-hour dark cycle.

### Space and density constraints

Following Molenaar *et al.* (*50*), we assume that the volume of the cell (*V*_tot_) is proportional to the total surface area (SA) occupied by transporters and lipids in the membrane multiplied by a factor β that corresponds to the volume–to–surface area ratio of the cell

$$V_{\mathrm{tot}}=(n_{\mathrm{tr}}s_{\mathrm{tr}}+n_{\mathrm{lm}}s_{\mathrm{lm}})\beta$$ (37)

in which *n*_tr_ and *n*_lm_ are the total number of transporters and lipids in the membrane, and *s*_tr_ and *s*_lm_ correspond to the specific surface area of transporters and lipid molecules, respectively. The number of molecules of a given intracellular pool *i* can be linked to their concentrations as follows

$$c_{i}=\frac{n_{i}}{V_{\mathrm{tot}}}$$ (38)

Thus, we can combine the equations above to get

$$1=(p_{\mathrm{tr}}s_{\mathrm{tr}}+c_{\mathrm{lm}}s_{\mathrm{lm}})\beta$$ (39)

Unsaturated lipids are expected to have higher *s*_lm_ relative to saturated lipids due to the presence of kinks in their molecular structure. Previous studies found that *s*_lm_ can be up to 1.25 times higher for unsaturated lipids versus saturated lipids (*59*, *60*). We performed sensitivity analyses assuming that *s*_lm_ decreases linearly with increases in temperature (due to increases in the saturation level of lipids; Eqs. 26 and 27) and found that this did not affect our main results (fig. S15). Thus, to minimize free parameters in the model, we decided to assume a constant value for *s*_lm_.

This formulation allows us to simplify the optimization problem to a single unknown parameter β. Because β is the volume-to-surface ratio of the cell, and assuming a spherical cell, we can also estimate cell size (radius, *r*) as

$$\beta =\frac{V_{\mathrm{tot}}}{\mathrm{SA}}=\frac{r}{3}$$ (40)

Note that other cellular geometries can be used with this model by modifying Eq. 40, but for simplicity, we use a sphere. We impose a constraint on the maximum allowable intracellular density of macromolecules *D*_max_ (Da μm^−3^). If the cell is at its maximum intracellular density and “needs” to allocate more resources to proteins (for example), then it would be required to also increase its cell size. To estimate the intracellular density of macromolecules, we must divide the abundance of intracellular components by the available volume. To do this, we divide the cellular volume into two reservoirs: the volume that is occupied by hydrophobic molecules (i.e., stored lipids) and organelles (i.e., photosystems) and the rest of the volume. It is this “other” volume *V* that is used to calculate the intracellular density of macromolecules (Eqs. 41 to 43). We can then write an expression for the total volume of the cell

$$V_{\mathrm{tot}}=V+(n_{p}+n_{\mathrm{dp}})V_{p}+(n_{\mathrm{li}}+n_{\mathrm{tag}})V_{\mathrm{li}}$$ (41)

in which *n*_p_ and *n*_dp_ are the total number of functional and damaged photosystems and *n*_li_ and *n*_tag_ are the total number of stored lipids required to regulate oxidative stress and to fuel respiration, respectively. *V*_p_ and *V*_li_ correspond to the volume of photosystem and stored lipid molecules, respectively. We can rewrite the expression above to obtain

$$\frac{V}{V_{\mathrm{tot}}}=1-(p_{p}+p_{\mathrm{dp}})V_{p}-(c_{\mathrm{li}}+c_{\mathrm{tag}})V_{\mathrm{li}}$$ (42)

This expression allows us to define the concentration of the pools that affect the intracellular density of macromolecules in the cell, which we denoted in Eq. 43 with subscript *k* and includes all pools in the model except for photosystems, lipid storage, and membrane components

$$\sum c_{k}\frac{1}{\eta_{k}\eta_{\mathrm{aa}}}\leq D_{\max}\frac{V}{V_{\mathrm{tot}}}$$ (43)

η*_k_* (number of amino acids per protein *k*) and η_aa_ (Daltons per amino acid) convert *c_k_* from units of molecule of protein per total cellular biovolume to Daltons per total cellular biovolume (table S2).

### Model validation and analyses

Our modeling predictions were compared against physiology, transcriptomic, proteomic, and metabolomic datasets compiled from the literature. Physiology data were available across entire temperature growth curves and allowed us to compare our modeling predictions against growth rates, carbon fixation rates, carbon use efficiency (i.e., 1 − respiration/photosynthesis), cell size (biovolume), cellular nitrogen-to-carbon ratio, and chlorophyll content per unit of biovolume. Whenever needed, we used the free version of GetData Graph Digitizer to digitize plots. ‘Omic datasets, however, commonly reported metabolic shifts between two temperatures, and thus, we were only able to perform qualitative comparisons for these data types (Table 1).

Physiology data were compiled from different sources for small (*Prochlorococcus*) and large (diatoms) phytoplankton acclimated to different temperatures under controlled experimental conditions (table S5). A total of six models were fit to the different datasets (table S3). Specifically, we tuned a subset of 10 model parameters to fit the models to the different datasets (table S3); all other parameter values were kept constant across all simulations and are given in table S2. The model parameters were kept within the range of values reported in the literature (see details for each parameter in Supplementary Text) while also capturing key differences in the observed thermal curves. For example, *E*_a_ and *T*_d_ values used for the simulations presented here (table S3) are within the range reported by Barton *et al.* (*10*). To simulate the higher protein content observed for *Chaetoceros* sp. (*29*) compared to *T. pseudonana* (*13*), *D*_max_ was increased for the simulations compared against Liang et al. (*29*). Similarly, on the basis of literature values of the volume of photosystems for cyanobacteria (Bionumber ID: 103908 and 103909) relative to diatoms (*61*), *V*_p_ was decreased for the small phytoplankton simulations. Last, the least well-constrained parameter in the model is the maximum allowed transporter–to–membrane lipid ratio (*M*_max_). To minimize the impact of this uncertainty on our conclusions, we performed a sensitivity analysis to confirm that changing this parameter does not alter our main findings (fig. S16).

We validated the model assuming that the cell could only use glycolysis (Fig. 2) or lipid degradation (fig. S1) to fuel dark respiration. This was done because our model solves for the optimal proteome of the cell in a steady-state environment and thus results in a sharp transition between the two metabolic pathways (it is never optimal to use both pathways). The tuned parameters for the model runs in which the cell could only use the glycolysis and the lipid degradation pathways are given in tables S3 and S4, respectively.

Often conversion factors were necessary to compare the model output to experimental data. The simulated pool of photosystems was converted to units of chlorophyll per biovolume assuming that one photosystem has, on average, 140 molecules of chlorophyll (Bionumber ID: 111790) (*62*) and considering that one molecule of chlorophyll has 893.5 g/mol. The carbon fixation rates reported in (*13*) were converted from units of μgC μgC^−1^ day^−1^ to μgC μm^−3^ day^−1^ assuming a constant cellular carbon content of 1.25 × 10^−4^ pmol C μm^−3^ [figure 3b in (*13*)].

We solved the model from 5° to 45°C in intervals of 1°C. We also performed a large number of sensitivity analyses to investigate (i) how the thermal dependencies of membrane components influence cell size (Fig. 4 and fig. S10), (ii) different protein damage formulations (fig. S5), (iii) the impact of poorly constrained parameters on traits and proteome investments (figs. S13 to S16), (iv) how traits change as the cell shifts from using glycolysis to using lipid degradation to fuel dark respiration (fig. S7), and (v) what influences the critical point at which the metabolic switch between glycolysis and lipid degradation occurs (fig. S8). In addition, we tested two different strategies through which a cell can invest resources to mitigate oxidative stress (i.e., stored lipids and oxidative enzymes; fig. S12) and the impact of different parameters on the possible adaptive strategies of phytoplankton (fig. S11). The model was written in Julia (*63*), and the code is deposited on Zenodo (DOI: https://doi.org/10.5281/zenodo.8125732) and is available on GitHub (https://github.com/LevineLab/ProteomePhyto/tree/main).

## Supplementary Materials

### This PDF file includes:

Supplementary Text Figs. S1 to S16 Tables S1 to S15 References
