# Supplementary material for: Mechanistic constraints on the trade-off between photosynthesis and respiration in response to warming
Source: Sci Adv. 2023 Sep 1;9(35):eadh8043. doi: 10.1126/sciadv.adh8043 (PMC10796116; doi:10.1126/sciadv.adh8043)
Supplement: Supplementary file 1 — Supplementary Text Figs. S1 to S16 Tables S1 to S15 References [file sciadv.adh8043_sm.pdf]

Science Advances
AAAS

Supplementary Materials for

Mechanistic constraints on the trade-off between photosynthesis and respiration in response to warming

Suzana G. Leles *et al.*

Corresponding author: Suzana G. Leles, leles@usc.edu; Naomi M. Levine, n.levine@usc.edu

Sci. Adv. **9**, eadh8043 (2023)
DOI: 10.1126/sciadv.adh8043

This PDF file includes:

Supplementary Text
Figs. S1 to S16
Tables S1 to S15
References

Supplementary Text

Supplementary Methods

The model is fully defined below. A list of the optimized variables is given in Table S1. Detailed description for each of the metabolic pathways are described in the main text of the paper.

C1-C8: Steady-state growth of proteins

$$\frac{d(p_p + p_{dp})}{dt} = \phi_p v_{ri} \frac{1}{\eta_p} - \mu(p_p + p_{dp}) = 0$$
$$\frac{dp_{ru}}{dt} = \phi_{ru} v_{ri} \frac{1}{\eta_{ru}} - \mu p_{ru} = 0$$
$$\frac{dp_{tr}}{dt} = \phi_{tr} v_{ri} \frac{1}{\eta_{tr}} - \mu p_{tr} = 0$$
$$\frac{dp_{ri}}{dt} = \phi_{ri} v_{ri} \frac{1}{\eta_{ri}} - \mu p_{ri} = 0$$
$$\frac{dp_{gl}}{dt} = \phi_{gl} v_{ri} \frac{1}{\eta_{gl}} - \mu p_{gl} = 0$$
$$\frac{dp_{lb}}{dt} = \phi_{lb} v_{ri} \frac{1}{\eta_{lb}} - \mu p_{lb} = 0$$
$$\frac{dp_{ld}}{dt} = \phi_{ld} v_{ri} \frac{1}{\eta_{ld}} - \mu p_{ld} = 0$$
$$\frac{dp_{re}}{dt} = \phi_{re} v_{ri} \frac{1}{\eta_{re}} - \mu p_{re} = 0$$

C9-C12: Steady-state growth of other macromolecules

$$\frac{dc_{ic}}{dt} = v_{ru} - v_{ri}\eta_{aac} - v_{lb} - v_{gl} - \mu c_{ic} = 0$$
$$\frac{dc_{in}}{dt} = v_{tr} - v_{ri}\eta_{aan} - \mu c_{in} = 0$$
$$\frac{dc_{lm}}{dt} = \alpha_{lm} v_{lb} \frac{1}{\eta_{lic}} - \mu c_{lm} = 0$$
$$\frac{dc_{tag}}{dt} = \alpha_{tag} v_{lb} \frac{1}{\eta_{lic}} - \mu c_{tag} = 0$$

C13: Proteome investments, in which i represents protein pools

$$\sum \phi_i = 0.5$$

C14: Fractions of the lipid synthesis flux:

$$\alpha_{lm} + \alpha_{tag} + \alpha_{ld} = 1.0$$

C15: Fraction of damaged photosystems (a function of heat damage and repair)

$$\frac{p_{dp}}{p_{dp} + p_p} = \frac{k_d p_p \frac{c_{tag_{max}}}{c_{tag}}}{k_{re} p_{re}}$$

C16: Constraint on the maximum concentration of stored lipids that mitigate damage

$$c_{tag} \leq c_{tag_{min}} + c_{tag_{max}}$$

C18: Membrane integrity

$$\frac{p_{tr}}{c_{lm}} \leq M_{min} + M_{max} \gamma_{Tm}$$

C19: Lipid degradation:

$$v_{ld} = \alpha_{lb} v_{lb}$$

C20-22: Energy metabolism

$$e_p v_p \geq e_{ru} v_{ru}$$

$$e_{cost} \leq (e_p v_p - e_{ru} v_{ru})$$

where, $e_{cost} = e_{tr}v_{tr} + e_{ri}v_{ri} + e_{lb}v_{lb} + e_{d}v_{d} + e_{f}v_{re}$

$f_{dr}e_{cost} \leq e_{gl}v_{gl} + e_{ld}v_{ld}$

C23: Space constraint

$1 = (p_{tr}s_{tr} + c_{lm}s_{lm})\beta$

C24: Density constraint, in which k indicates all pools of the cell except for photosystems, lipid storage and membrane components

$$\sum c_k \frac{1}{\eta_k} \leq D_{max} \frac{V}{V_{tot}}$$

where, $\frac{V}{V_{tot}} = 1 - (p_p + p_{dp})V_p - (c_{li} + c_{tag})V_{li}$

Reaction rates are defined as following:

$$v_p = k_{ref_p} p_p \frac{I}{I + K_p}$$
$$v_{ru} = k_{ref_{ru}} \gamma_{Ta} p_{ru} \frac{DIC}{DIC + K_{ru}}$$
$$v_{tr} = k_{ref_{tr}} \gamma_{Ta} p_{tr} \frac{DIN}{DIN + K_{tr}}$$
$$v_{ri} = k_{ref_{ri}} \gamma_{Ta} p_{ri} \frac{c_{ic}}{c_{ic} + K_{ic}} \frac{c_{in}}{c_{in} + K_{in}}$$
$$v_{gl} = k_{ref_{gl}} \gamma_{Ta} p_{gl}$$
$$v_{lb} = k_{ref_{lb}} \gamma_{Ta} p_{lb} \frac{c_{ic}}{c_{ic} + K_{lb}}$$
$$v_{ld} = k_{ref_{ld}} \gamma_{Ta} p_{ld}$$
$$v_d = k_{ref_d} \gamma_{Td} p_p \frac{c_{tag_{max}}}{c_{tag}}$$
$$v_{re} = k_{ref_{re}} \gamma_{Ta} p_{re} \frac{p_{dp}}{p_{dp} + p_p}$$

Temperature effects:

$$\gamma_{Ta} = exp(\frac{E_a}{R}(\frac{1}{T_{ref}} - \frac{1}{T}))$$
$$\gamma_{Td} = exp(\frac{E_d}{R}(\frac{1}{T_d} - \frac{1}{T}))$$
$$\gamma_{Tm} = (\frac{T_{max} - T}{T_{max} - T_{min}})$$

Number of molecules of nitrogen per amino acid:

$$\eta_{aan} = \eta_{aac} \sum \phi_i q_i$$

Estimated storage pools that fuel dark respiration:

$$c_{gu} = v_{gl}\frac{1}{\eta_{guc}}td$$
$$c_{li} = v_{ld}\frac{1}{\eta_{lic}}td$$

Cell nitrogen to carbon quota (q_{cell}), in which i represents protein pools and η_i represents the molecular weight of proteins in units of aa protein^{-1}:

$$q_{cell} = \frac{\sum c_i\eta_i\eta_{aac}q_i + c_{in}}{\sum c_i\eta_i\eta_{aac} + c_{ic} + (c_{lm} + c_{li} + c_{tag})\eta_{lic} + c_{gu}\eta_{guc}}$$

Parameter values

All parameter values can be found in Tables S2 and S3. Detailed description for the choice of parameter values can be found in the next sections.

Enzymatic kinetics

All maximum turnover rates obtained from the literature were corrected for a reference temperature of 20 °C when necessary assuming a Q_{10} value equal to 2. The only exception was photochemistry which is assumed to be temperature independent (*23*). If rates specific for phytoplankton were not found in the literature, we used enzyme kinetics obtained from the BRENDA database. Specific details on the different k_{ref} values are given below.

k_{ref_p}: according to (*31*), PQH_2 is the rate limiting step in photosynthetic electron transfer, being 5 to 8 times slower than the rate of photon capture which approaches 1.2 photons per ms per PSII. According to (*51*), the rate limiting step in photosynthesis is NAH-1 (130 s^{-1}). Thus, the maximum rate of photon absorption by photosystems is between 7800-9000 min^{-1}. We assumed k_{ref_p} to be 7800 min^{-1} in our simulations.

$k_{ref_{ru}}$: the maximum turnover rate of rubisco was estimated as 126-222 min^{-1} at 25 °C for different diatom

species (*64*). Assuming a Q_{10} equal to 2 we obtain 89-157 min^{-1} at 20 °C. We assumed $k_{ref_{ru}}$ to be 157 min^{-1} in our simulations.

$k_{ref_{ri}}$: the translation rate of amino acids by ribosomes was estimated as 114 min^{-1} at 20 °C for the diatom *Thalassiosira weissflogii* (*53*).

$k_{ref_{gl}}$: assuming that the rate limiting step in glycolysis is phosphofructokinase, the maximum turnover rate can vary between 5700 min^{-1} at 50 °C (*65*) to 390 min^{-1} at 37 °C (*66*) (BRENDA:EC2.7.1.11), which after correcting for temperature gives us the range of 120-712 min^{-1} at 20 °C. We assumed $k_{ref_{gl}}$ to be 120 min^{-1} in our simulations.

$k_{ref_{lb}}$: assuming that the rate limiting step in lipid synthesis is acetyl-Coa carboxylase, the maximum turnover rate can vary between 596 min^{-1} at 37 °C (*67*) to 696 min^{-1} at 20 °C (*68*) (BRENDA:EC6.4.1.2), which after correcting for temperature gives us the range of 114-696 min^{-1} at 20 °C. We assumed $k_{ref_{lb}}$ to be 120 min^{-1} and equal to $k_{ref_{gl}}$.

$k_{ref_{ld}}$: assuming that the rate limiting step in fatty acid oxidation is acyl-CoA dehydrogenase, the maximum turnover rate can vary between 900 min^{-1} at 70 °C (*69*) to 2700 min^{-1} at 25 °C (*70*) (BRENDA:EC1.1.1.35), which after correcting for temperature gives us the range of 28-1900 min^{-1} at 20 °C. We assumed $k_{ref_{ld}}$ to be 120 min^{-1} and equal to $k_{ref_{gl}}$.

$k_{ref_{tr}}$: this value is not well constrained in the literature thus we assumed the maximum transport rate of nitrogen per transporter to be the same as $k_{ref_{gl}}$, $k_{ref_{lb}}$, $k_{ref_{ld}}$, i.e. 120 min^{-1} at 20 °C.

$k_{ref_{re}}$: diatoms are known to quickly repair photo-damaged photosystems (*71*). The repair rate of proteins is estimated to be fast and approximately 1-2 orders of magnitude faster than protein synthesis (*57*). Thus, we assumed a repair rate of 1000 min^{-1} at 20 °C, being one order of magnitude faster than $k_{ref_{ri}}$.

$k_{ref_{d}}$: we assumed that damage-repair dynamics are fast relative to other processes such as protein synthesis following (*26*) and set the damage rate to be equal to the repair rate, i.e. 1000 min^{-1} at 20 °C.

Temperature effects and space constraints

Parameters related to temperature effects (i.e. E_a, E_d, T_d, T_{min} and T_{max}) were based on the experimentally derived rates from (*10*). The choices for the values of parameters influencing space constraints are given below:

s_{tr}: the transporter complex radius is 10^{-3} μm (*53, 72*)]. Considering that the surface area of a sphere is equal to $4\pi R^2$, we obtain that the surface area of a molecule of transporter is equal to 1.26 $\times$ $10^{-5}\mu m^2$.

s_{lm}: the average surface area occupied by a lipid molecule is 0.4-0.5 $\times$ $10^{-6}\mu m^2$ [BNID 114186, (*73*)]

M_{min} and M_{max}: the minimum and the maximum ratios between transporter and lipid molecules in the membrane, respectively, are not well constrained by the literature. Therefore, we performed sensitivity analyses to evaluate model behavior when changing these parameters (Figure S16).

V_p: the average volume occupied by a photosystem molecule is 3 $\times$ $10^{-5}\mu m^3$ for *Thalassiosira pseudonana* (*61*) and between 1 $\times$ 10^{-6} - 2 $\times$ 10^{-5} μm^3 for the cyanobacteria *Synechococcus* (BioNumbers: ID103908 and ID103909).

V_{li}: we use tripalmitin as our modelled TAG molecule due to its abundance in marine systems. Tripalmitin has a density of 0.875 g cm^{-3}, which converting to mass (multiplying by the Avogadro number; 6.02 $\times$ 10^{23}) and to μm^{-3} (dividing by 10^{12}) gives us 5.3 $\times$ 10^{11} Da μm^{-3}. Considering that a 16C TAG molecule has a molecular weight of 800 Da, we estimate the volume of a TAG molecule to be 1.5 $\times$ $10^{-9}\mu m^3$.

D_{max}: the maximum density of protein in *Chaetoceros* sp. has been estimated to be 110 fg protein μm^{-3} (*29*). Assuming that half of the proteome is constant (see p_{other} below) and that on average 32.2% of the cell dry weight is protein (*74*), we estimate the total maximum intracellular density of macromolecules of the cell to be 341 fg μm^{-3}, which converting to grams (dividing by 10^{15}) and to mass (multiplying by the Avogadro number) gives us 2.0 $\times$ 10^{11} Da μm^{-3}.

p_{other}: the maximum density of the other proteome fraction not simulated was assumed to be half of the total protein content estimated by (*29*), i.e. 55 fg protein μm^{-3}, which converting to grams (dividing by 10^{15}) and to mass (multiplying by the Avogadro number) gives us 3.3 $\times$ 10^{10} Da μm^{-3}, which is approximately 5 times smaller

than D_{max} (see Figure S13 for sensitivity analysis).

$c_{tag_{max}}$: the maximum concentration of lipid storage in the cell (not used to fuel dark respiration) was constrained based on the average fraction of the cellular volume that is occupied by lipids, i.e. 20% (*74*), resulting in a value of 2×10^7 molecules μm^{-3}. We also set a minimum concentration of lipid storage $c_{tag_{min}}$ to avoid damage going to zero and chose a low value of 200 molecules μm^{-3}.

Molecular weights

The molecular weight η of each protein pool was obtained from the UniProt database using the diatom species *Fragilariopsis cylindrus* as a model species. The total molecular weight of a given modeled protein pool correspond to the sum of all entries retrieved in the database for a given pathway (see below) following (*52*).

η_p: 999025 Da or 9082 aa/photosystem; search = photosystem (Table S6).

η_{ru}: 652716 Da or 5933 aa/rubisco; search = rubisco (Table S7).

η_{tr}: 115049 Da or 1046 aa/transporter; search = nrt2 transporter (Table S8).

η_{ri}: 2133276 Da or 19393 aa/ribosome; search = ribosomal proteins, including 30S, 50S, 40S and 60S (Tables S9 and S10).

η_{gl}: 1674239 Da or 15220 aa/gl; search = glycolysis (Table S11).

η_{lb}: 1511627 Da or 13742 aa/lb; search = lipid synthesis which includes glycerolipid, glycolipid, membrane lipid metabolisms and acetyl-Coa carboxylase (Table S12).

η_{ld}: 564133 Da or 5128 aa/ld; search = fatty acid beta oxidation (Table S13).

η_{re}: 698544 Da or 6350 aa/re; repair proteins were identified according to (*75*); search = chaperone, disassembly PSII, D1 phosphorylation, degradation photodamaged D1, assembly D1 (Table S14).

η_{lic}: we assume a total of 16 carbon molecules per molecule of lipid storage and membrane lipid. This value was chosen based on the most abundant TAG and phospholipid molecules in the ocean, tripalmitin (16:0/16:0/16:0) and phosphatidylglycerol (16:0/16:1), respectively [see Figure 2 in (*15*)]. This is in agreement with the mean chain C length of fatty acids observed for diatoms (*15*).

η_{li}: the molecular weight of a 16C lipid is approximately 800 Da (*76*).

According to Figure 1 in (*49*), the nitrogen to carbon quota of ribosomes is larger than that of proteins which in turn is larger than that of photosystems. Ribosomes are more enriched in nitrogen than an average protein because it contains RNA (*49*). While proteins are approximately 25% nitrogen and 75% carbon, one molecule of chlorophyll has 4 atoms of nitrogen and 55 atoms of carbon, which results in a lower nitrogen to carbon quota. Thus, here we assumed the following nitrogen to carbon quotas for ribosomes, proteins and photosystems, respectively: $q_{ri} = 0.33$, $q_{pt} = 0.20$ and $q_p = 0.10$.

Energy conversion factors

The energy conversion factors are given in ATP units, assuming that 1 mol of NADPH is equivalent to 2.6 moles of ATP.

e_{tr}: 1 ATP is required to import 1 molecule of nitrogen into the cell following (*53*).

e_p: considering the energetic stoichiometry of core photosynthetic processes, one ATP is produced per photon energising PSI (*77*).

e_{ru}: assuming no loss processes in the carbon concentration mechanisms, the energy cost to convert CO_2→carbohydrate is between 9.25-10 molecules of ATP per carbon (*31, 77*). Here we set this value as 10 molecules of ATP per carbon.

e_{lb}: (*31*) estimated that the synthesis of lipids from CO_2 requires 6.3 ATP + 2.9 NADPH, totaling in 13.9 molecules of ATP per carbon. We model lipid synthesis in two steps, i.e. $CO_2 \rightarrow$carbohydrates (e_{ru}) and carbohydrates$\rightarrow$lipids (e_{lb}). Therefore, e_{lb} can be estimated as 13.9 minus 10 = 3.9 molecules of ATP per carbon.

e_{gl}: Glucose oxidation via glycolysis and the citric acid cycle recovers 5 molecules of ATP per carbon (*31*).

e_{ld}: fatty acid oxidation via the β-oxidation pathway and the citric acid cycle provides an energy recovery of 6.6 molecules of ATP per carbon (*31*).

e_{ri}: the energy cost associated to the protein synthesis flux was estimated considering that a total of 45 molecules of ATP are required in the metabolic pathway to synthesize one amino acid and that 3 molecules of ATP are consumed for one translation elongation step (*52, 78*), giving a total of 48 molecules of ATP per amino acid.

e_{re}: we assumed the energy cost of repairing heat-damaged photosystems to be the same as that of repairing photo-damaged photosystems. The repair energy cost has been estimated to be 0.8-19% of net protein synthesis in growth among microbes (*79*) and 1.7-4.6% of the ATP required for the gross carbon assimilation in plants (*80*). Thus, we can estimate the repair energy cost to vary between 0.16-9 molecules of ATP per photosystem; here we assumed an averaged value of 4.5 molecules of ATP per photosystem.

e_d: we assumed that the damage rate of photosystems due to heat has the same cost as the repair of photosystems when $c_{tag_{max}} = 2 \times 10^7$ molecules μm^3 and that it scales with $c_{tag_{max}}$ as following: $e_d = \frac{9\times10^7}{c_{tag_{max}}}$.

Supplementary Results

Photosystems damage-repair

Following (*26*), we assumed that the damage-repair dynamics of photosystems converges to a quasi steady-state because folding processes are fast compared to growth processes (such as protein synthesis). To test the sensitivity of this assumption, we explicitly simulate damage-repair dynamics and demonstrate that our quasi steady-state approach is robust. Specifically, we modify our model and explicitly simulate the pool of functional (p_p) and damaged

(p_{dp}) photosystems:

$$\frac{dp_p}{dt} = \phi_p v_{ri} \frac{1}{\eta_p} - v_d + v_{re} - \mu p_p = 0$$
$$\frac{dp_{dp}}{dt} = v_d - v_{re} - \mu p_{dp} = 0$$

We also modify the rate of repair to a Michaelis-Menten type equation based on the concentration of damaged photosystems p_{dp}:

$$v_{re} = k_{re} p_{re} \frac{p_{dp}}{p_{dp} + K_{re}}$$

By assuming that $K_{re} = 1000$, the number of functional and damaged photosystems are conserved among the two modeling approaches (Figure S4) and we find that the main results reported in our work do not change.

Model parameter sensitivity analyses

We tested the sensitivity of our model to the choice of parameter values that are not well constrained by the literature, specifically p_{other}, M_{max} and f_{dr} (Figures S13-S16). We also tested the sensitivity of the model to the pools that experience heat stress damage (Figure S5), and the thermal dependency of transporters (Figure S10). We also conducted sensitivity tests to evaluate how different parameters could impact the adaptive responses of phytoplankton that evolved at warm temperatures (Figure S11) and the impact of different lipid surface areas s_{lm} (Figure S15).

The baseline runs correspond to the results presented in Figure 2. The concentration of the proteome that is not being dynamically simulated in the model (p_{other}) does not change our main results, with little to no impact on growth rate, carbon use efficiency and the relative proteome investments (Figure S13). However, it can affect the pool of other macromolecules, biovolume and rates of carbon fixation quantitatively, since higher p_{other} means that there is less space available to other structures, which ultimately decreases rate capacity. We also evaluated the sensitivity of our results to the fraction of the total energetic cost that must be paid by respiration that happens in the dark (f_{dr}). Changes in f_{dr} do not change our results qualitatively, but it affects all variables quantitatively in similar proportions. This happens because higher f_{dr} requires higher investment in respiration rates and thus requires more space that is allocated to carbon storage, which decreases growth capacity and increases cell size (Figure S14).

Finally, the maximum transporter to lipid ratio in the membrane has a direct impact on cellular biovolume, with lower values resulting in smaller biovolumes, with no major impact in any other variables or our main conclusions (Figure S16).

Our baseline model assumes that only photosystems are damaged by heat stress. In reality, many protein pools will be denatured as temperatures increase. Including damage for all protein pools in the model decreases reaction rates, decreases carbon use efficiency, and increases respiration demands at critical high temperatures (Figure S5, purple versus green). However, interesting trade-offs in the model arise due to differences in relative investments. Specifically, in this case the trade-offs arise because photosystems are temperature independent while all other reactions are temperature dependent and because damage rates are higher for photosystems. We show that the model simulations in which damage impacts all protein pools results in the same trade-offs and investment patterns as our baseline model (as shown in Figure S5).

Metabolic switch simulations

We investigated the sensitivity of the metabolic switch between glycolysis and lipid degradation to model parameters. We systematically tested how changes in parameter values impacted the proteome investment in lipid degradation and the critical point at which the cell switches between respiratory pathways (Figure S8). We found that increases in the recoverable energy (e_{ld}) and the maximum turnover rate of lipid degradation (k_{ld}) results in a more efficient lipid respiratory pathway, driving the cell to switch to lipid degradation at lower temperatures and even decreasing the relative proteome requirements. Decreasing D_{max} results in less cellular space available for all macromolecules, which increases the advantage of the lipid degradation pathway since lipids take up less space than carbohydrates. Increases in the fraction of the total energetic cost that happens in the dark (f_{dr}) increases respiration rates and thus requires higher lipid synthesis to build more lipid storage that will fuel respiration. The metabolic switch is relative insensitive to changes in the thermal dependency of metabolic reactions (E_a), except if the E_a of respiration is lower than at least one other process in the model (e.g. ribosomes), which would require higher investment in respiration, favoring the switch to lipid degradation as temperature increases. Finally, the lipid degradation pathway is not sensitive to changes in the maximum number of transporters to lipids in the membrane (M_{max}).

Antioxidant enzyme simulations

We developed a version of the model in which the cell can choose to synthesize antioxidant enzymes to minimize heat-damage instead of storing lipids. This model includes two additional state variables: a protein pool of antioxidant enzymes p_{ox} and the relative proteome investment in antioxidant enzymes ϕ_{ox}. Thus, the constraint described in Equation 5 ($\sum \phi_i = 0.5$) is modified to also account for ϕ_{ox}. Similar to the process described for stored lipids, we assume that the cell can choose to maximize the concentration of antioxidant enzymes up to $p_{ox_{max}}$ to minimize the rate of damage of photosystems as temperatures increase, or it can choose to store less p_{ox} at the cost of higher damage, so that:

$$p_{ox} \leq p_{ox_{min}} + p_{ox_{max}}, \tag{44}$$

$$v_d = k_d p_p \frac{p_{ox_{max}}}{p_{ox}}, \tag{45}$$

in which the minimum concentration of antioxidant enzymes $p_{ox_{min}}$ is required to avoid v_d going to zero. The ODE equation that describes the p_{ox} pool can then be written as:

$$\frac{dp_{ox}}{dt} = \phi_{ox} v_{ri} \frac{1}{\eta_{ox}} - \mu p_{ox} = 0, \tag{46}$$

in which η_{ox} is the molecular weight of the antioxidant enzymes. Here, for simplicity, we assumed η_{ox} to be equal to the molecular weight of the constant pool of proteins that are not resolved by the model (p_{ot}).

The cell must accommodate intracellular space for antioxidant enzymes similar to the model version that considers stored lipids. A key difference, however, is that the cell only requires carbon to build lipids while it requires both carbon and nitrogen to build antioxidant enzymes. We show that this metabolic strategy for mitigating oxidative stress requires the cell to have higher N:C quota, but that the main conclusions remain unchanged (Figure S12). This analysis indicates that our findings are broadly applicable because resources devoted towards heat-stress mitigation (whether it is through lipid storage or some other process) diverts resources away from other cellular processes. The strength of the model is in helping elucidate where those trade-offs are and what drives the trade-off (e.g. the relative energy, space and/or resource requirements).

Figures

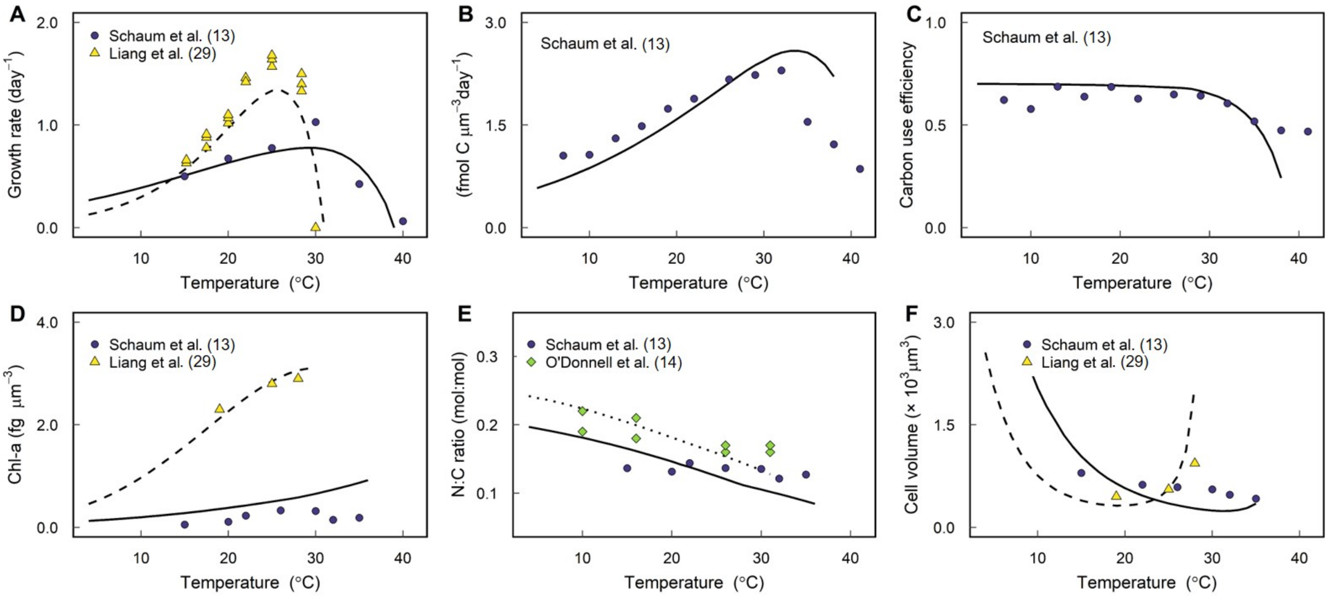

Figure S1. Model validation for thermal acclimation. Comparison between model (lines) and experimental data (symbols) for diatoms acclimated to different temperatures. Solid lines correspond to model calibrated to data from *Thalassiosira pseudonana* CCMP1335 (*13*), dashed lines to *Chaetoceros* sp. CCMP160 (*29*) and dotted line to *Thalassiosira pseudonana* CCMP1335 (*14*). Here we assume the cell can only invest in lipid degradation to fuel dark respiration but model validation was also performed assuming that the cell can only invest in glycolysis (Figure 2). Carbon use efficiency is unitless and is defined as 1 - respiration/photosynthesis where respiration and photosynthesis have units of $fmol$ C μm^{-3} day^{-1}. Panel B shows carbon fixation rates. For model parameterization see Table S4.

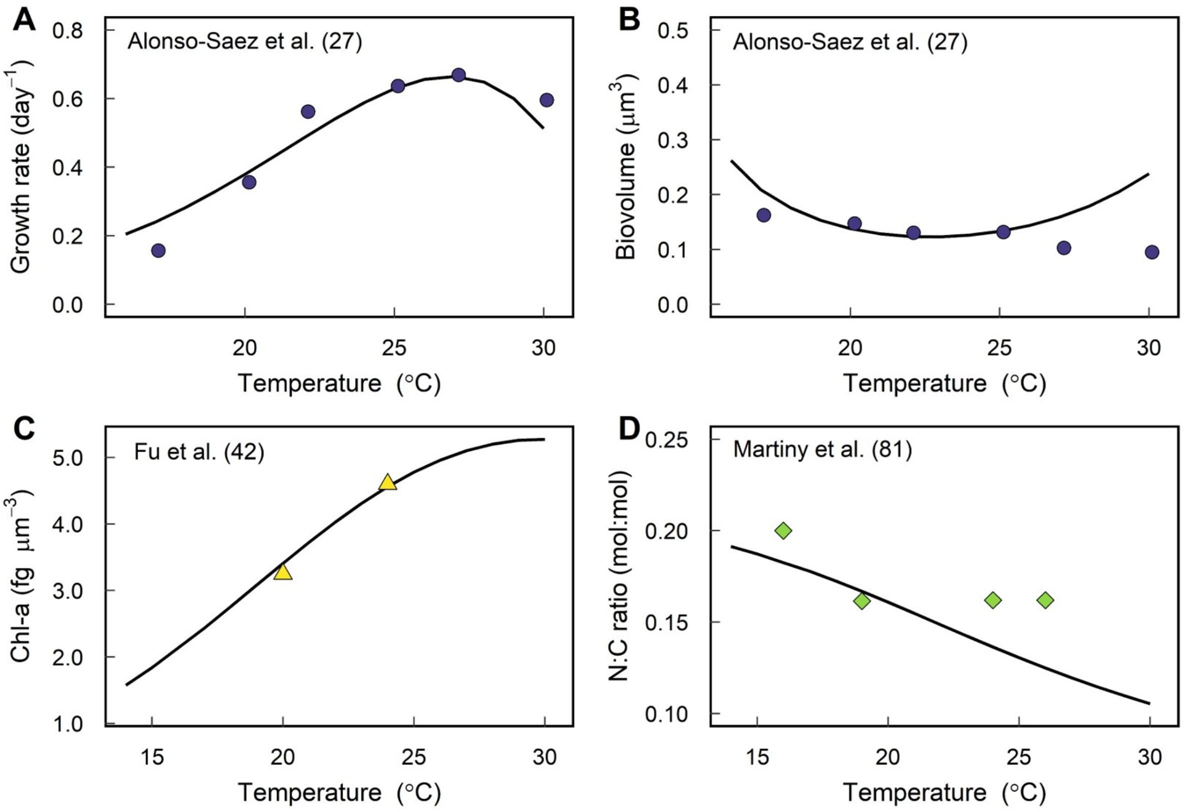

Figure S2. Model validation against cyanobacteria data. The model (lines) was compared against experimental data (symbols) obtained for different strains of *Prochlorococcus* acclimated at different temperatures. Different symbols and colors indicate the data sources as given within each panel. *Prochlorococcus* strains were as following for each study: *Prochlorococcus marinus* strain MIT9301 (*27*), CCMP1986 (*42*), and VOL7 derived from MED4 (*81*). Here we assumed the cell could only use glycolysis to fuel dark respiration. For model parameterization see Table S3.

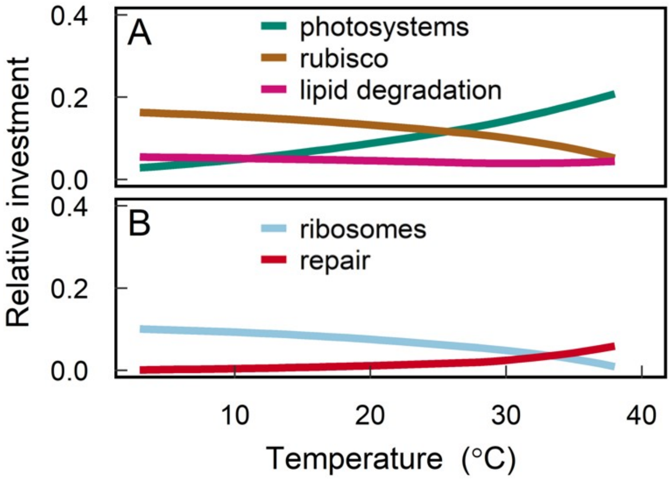

Figure S3. Optimal proteome investments. Changes in the relative proteome investment in different protein pools as a function of acclimated temperature. Here lipid degradation was the only respiratory pathway simulated. Model output is shown for the model calibrated according to (*13*).

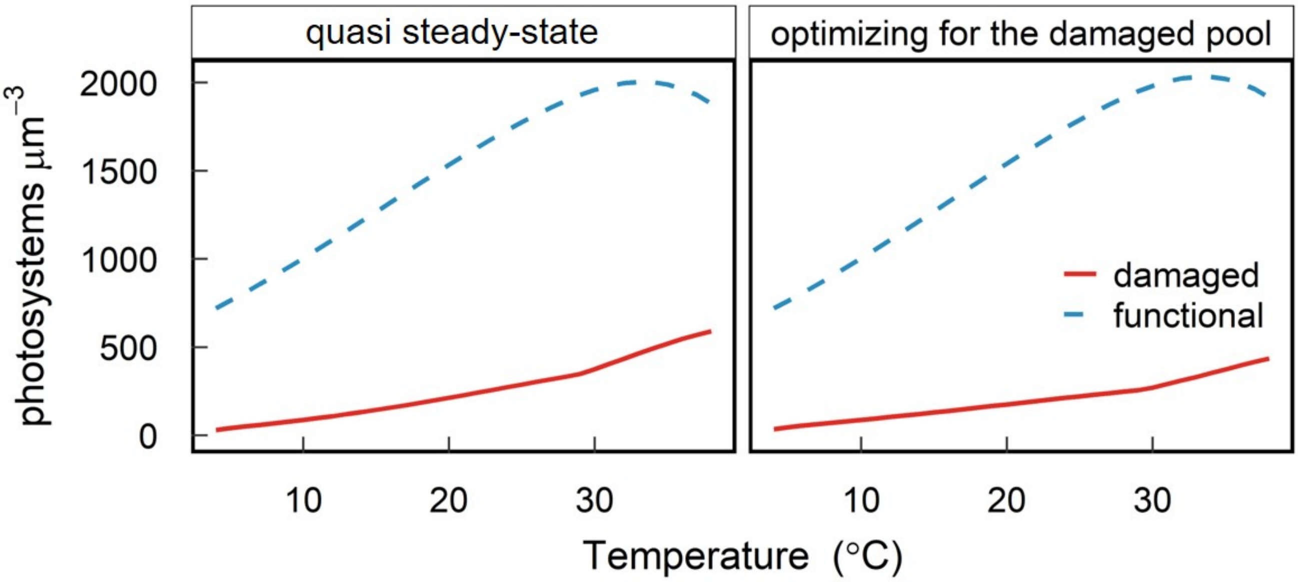

Figure S4. Photosystems as a function of temperature. Number of functional (dashed blue line) and damaged (solid red line) photosystems per unit of biovolume as a function of temperature. Results are shown for the quasi steady-state approximation following the damage-repair dynamics by (*26*) and for model runs in which the model optimizes for the damaged pool of photosystems (assuming $K_{re} = 1000$).

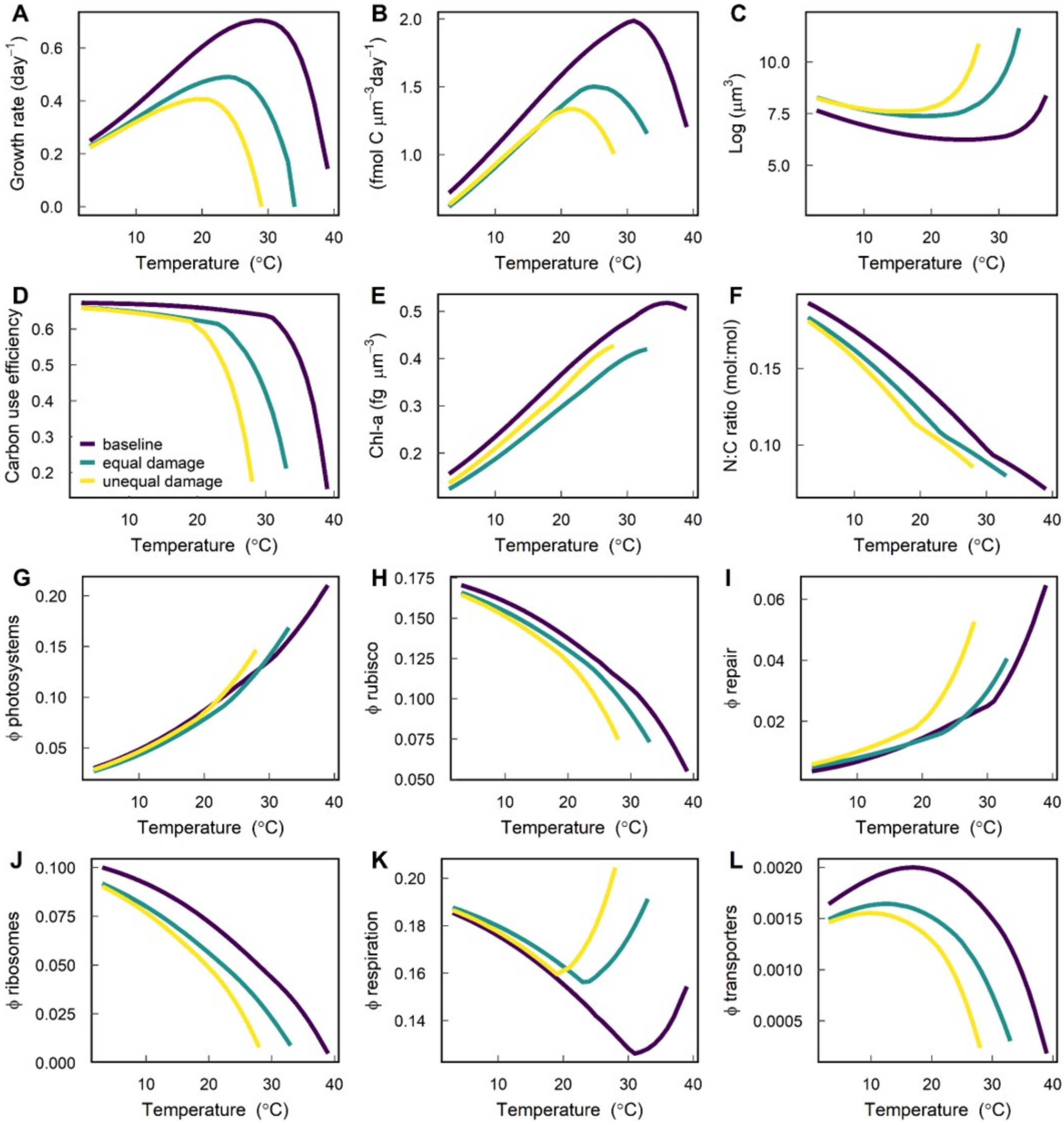

Figure S5. Sensitivity analysis to protein damage. Comparison between our baseline run that assumes that only photosystems are damaged by temperature (purple lines; Figure 2) versus a version of the model where all proteins are assumed to be equally damaged by temperature (green lines) and a version in which photosystem damage is assumed to be greater than for the other proteins (yellow lines).

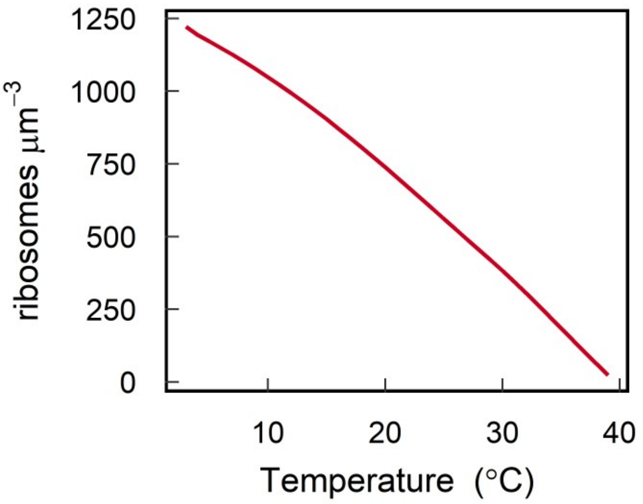

Figure S6. Ribosomes as a function of temperature. Number of ribosomes per unit of biovolume as a function of temperature.

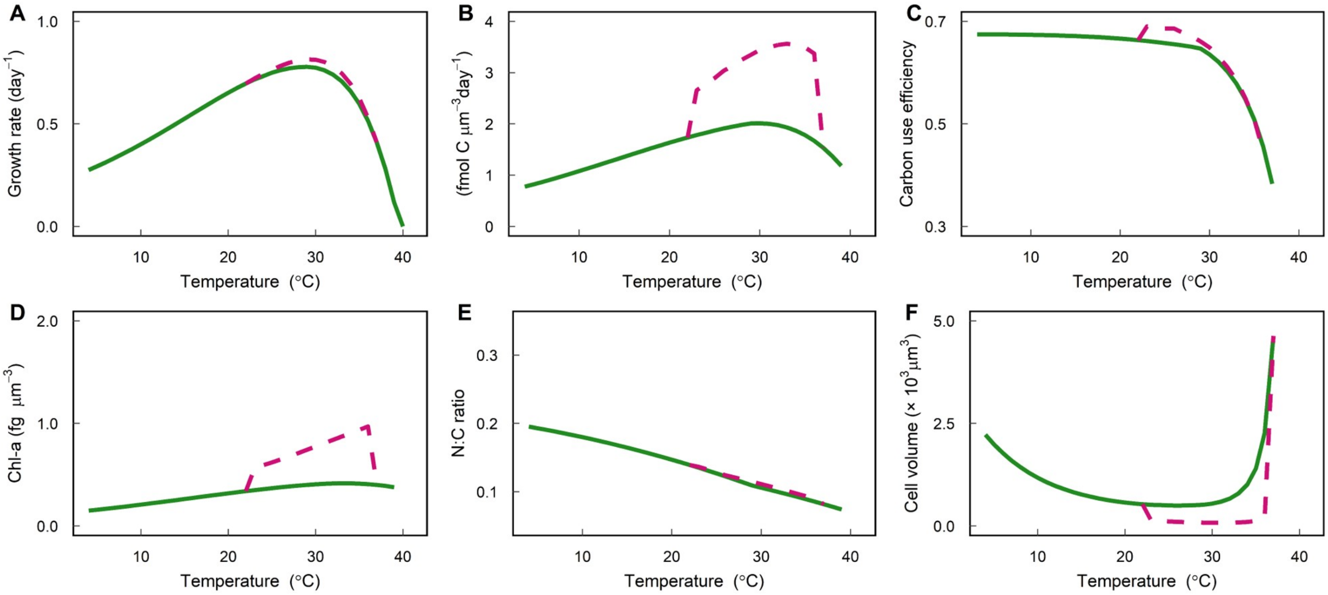

Figure S7. Trait changes as the cell switches between respiratory pathways. When given the choice between both respiratory pathways, the cell switches from glycolysis (solid green lines) to lipid degradation (dashed pink lines) once temperatures pass a critical point.

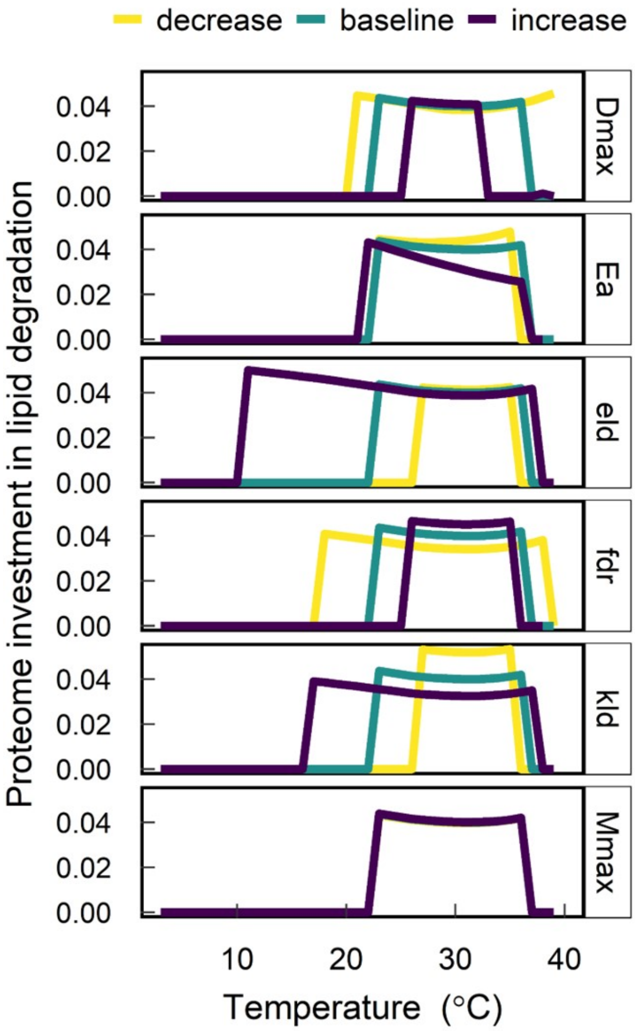

Figure S8. Sensitivity analysis on the lipid degradation pathway. Sensitivity tests were performed to evaluate how different parameters (shown by the different panels) influence the critical point in which the cell chooses to invest in the lipid degradation pathway. Parameters were increased and decreased relative to the baseline value used in Figure 3C (Table S15).

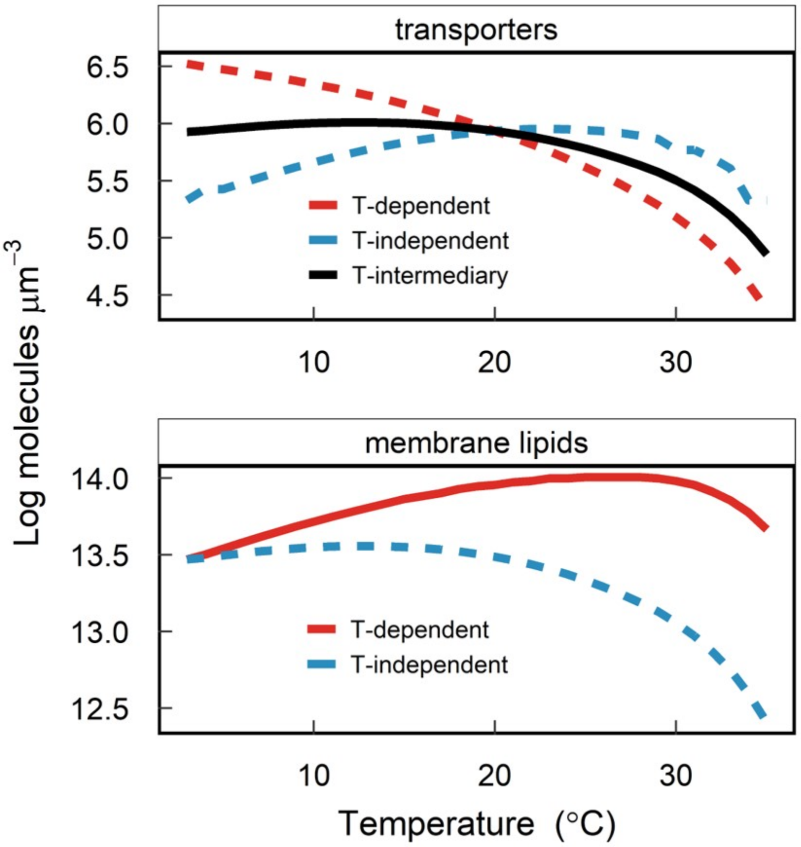

Figure S9. Membrane components as a function of temperature. Changes in the concentration of nutrient transporters (A) and membrane lipids (B) as a function of temperature. Solid lines indicate the baseline run (shown in Figures 2 and 3) while dashed lines indicate the sensitivity runs. In panel A, the T-dependent run (red dashed) uses the same temperature sensitivity for transporters as other reactions. To provide the other extreme case, runs in which transporters are assumed to be independent of temperature are shown (blue dashed). The baseline uses an intermediary scenario for nutrient transporters (see Figure 1B).

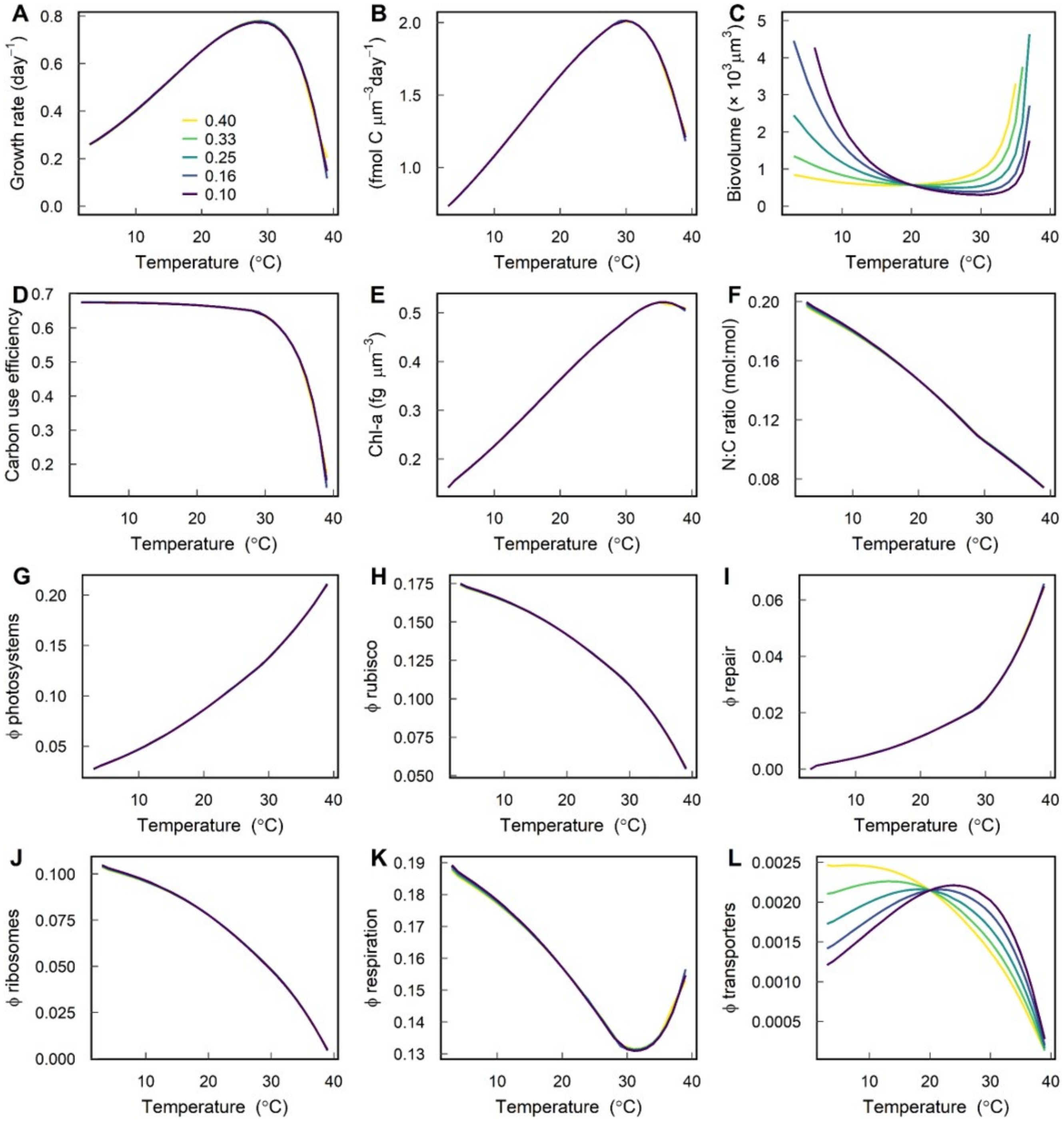

Figure S10. Sensitivity analysis to the thermal dependency of transporters. The E_a value of transporters was assumed to be lower than the E_a of other reactions which was set at 0.5. Our baseline run assumed that the E_a of transporters was equal to 0.25.

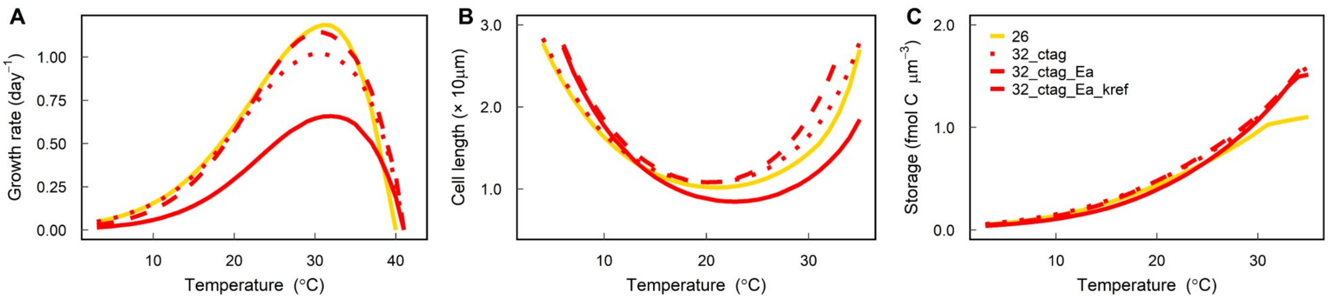

Figure S11. Sensitivity tests to evaluate thermal adaptation under severe warming. Adaptation to moderate warming (26°C; yellow) was simulated by increasing the activation energy of enzymatic reactions E_a (Table S3). Adaptation to severe warming (32°C; red) was simulated by increasing both the maximum lipid content $ctag_{max}$ and E_a as well as decreasing the maximum turnover rate of all reactions k_{ref} (Table S3). Here we show the potential adaptive effects of only changing $ctag_{max}$ (dotted red lines), $ctag_{max}$ and E_a (dashed red lines), and all three parameters (solid red lines; same as in Figure 5). In panel C, carbon storage accounts for both carbohydrates and lipids required to meet dark respiration costs as well as stored lipids to regulate oxidative stress.

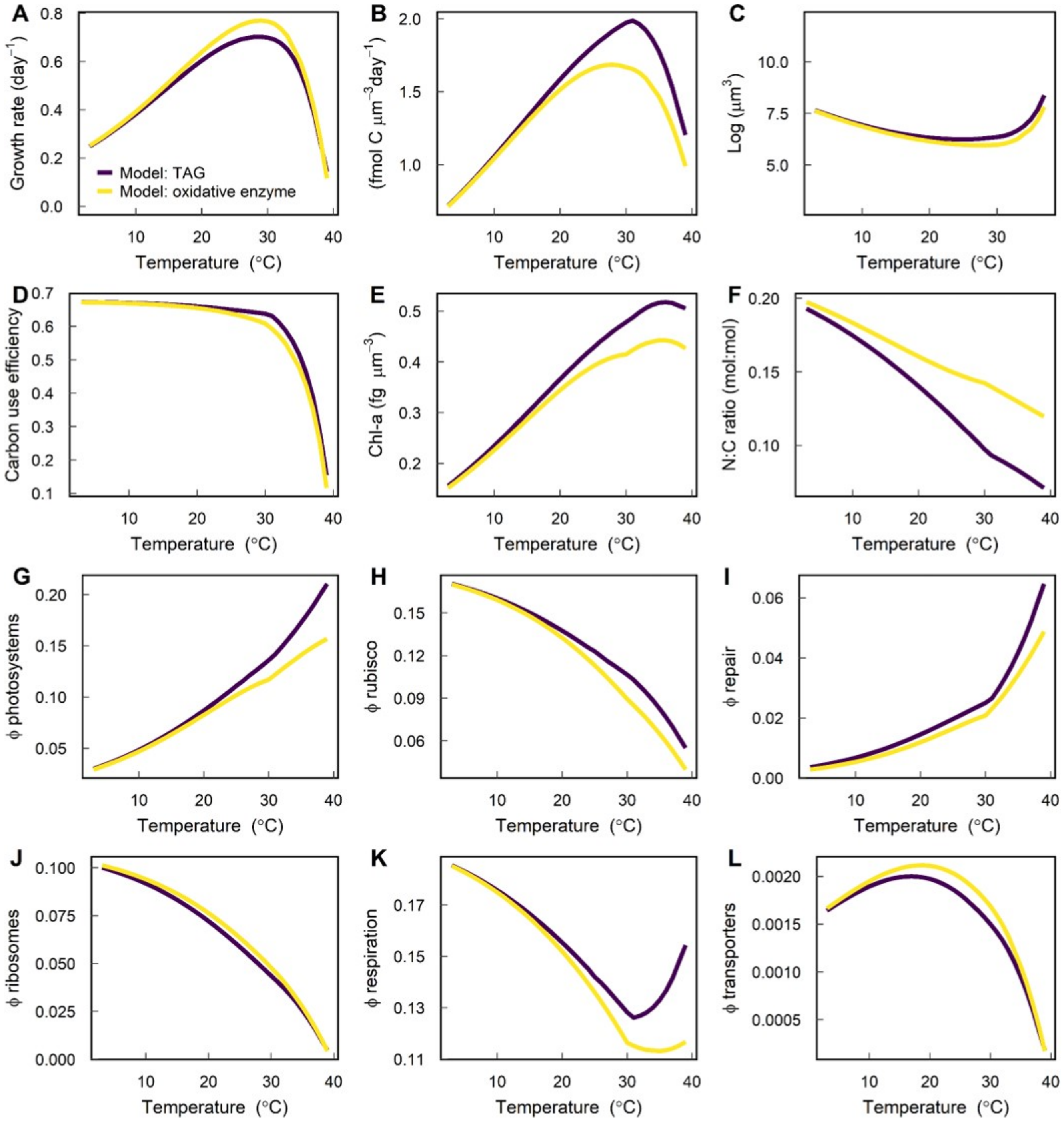

Figure S12. Different strategies to minimize heat damage. The baseline model assumes the cell can store TAGs to minimize heat-damage. The alternative model assumes the cell can synthesise antioxidant enzymes to minimize heat-damage. While the synthesis of lipids requires only carbon, the synthesis of antioxidant enzymes requires both carbon and nitrogen.

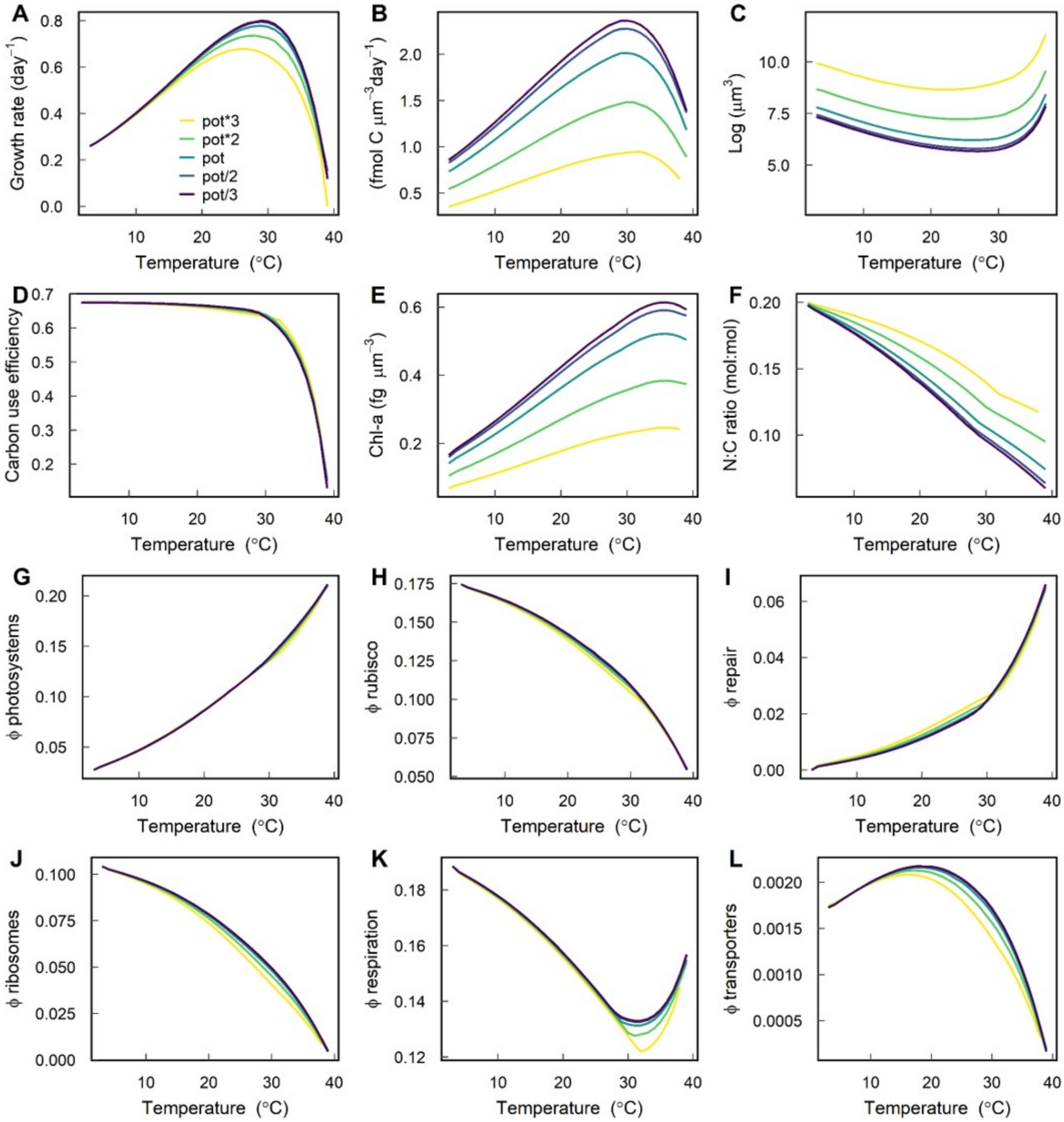

Figure S13. Sensitivity analysis to p_{other}. This parameter sets the concentration of other proteome not included in the model. ϕ indicates relative proteome allocation in a given protein.

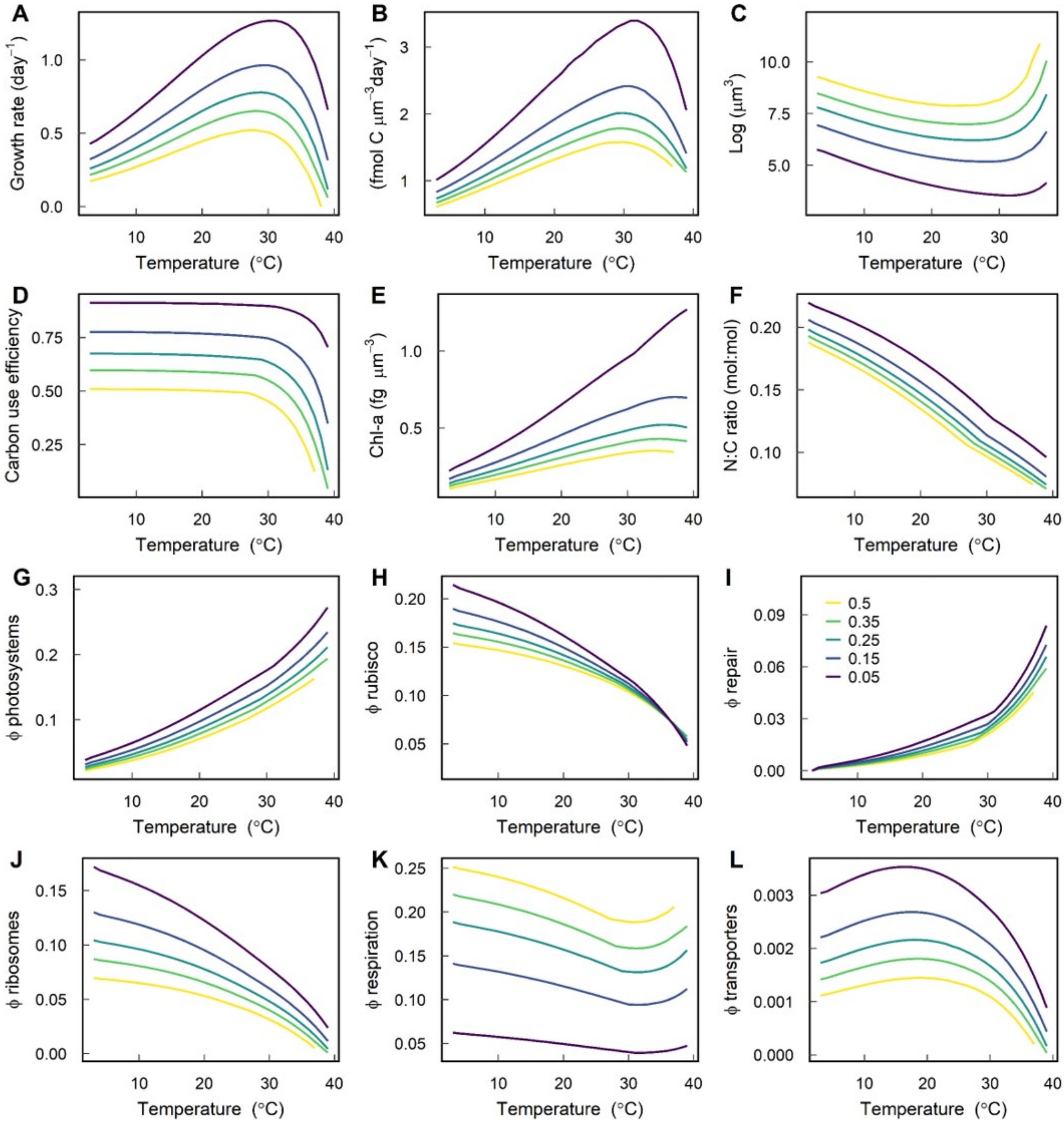

Figure S14. Sensitivity analysis to f_{dr}. This parameter sets the fraction of the total energetic costs that happens in the dark. ϕ indicates relative proteome allocation in a given protein.

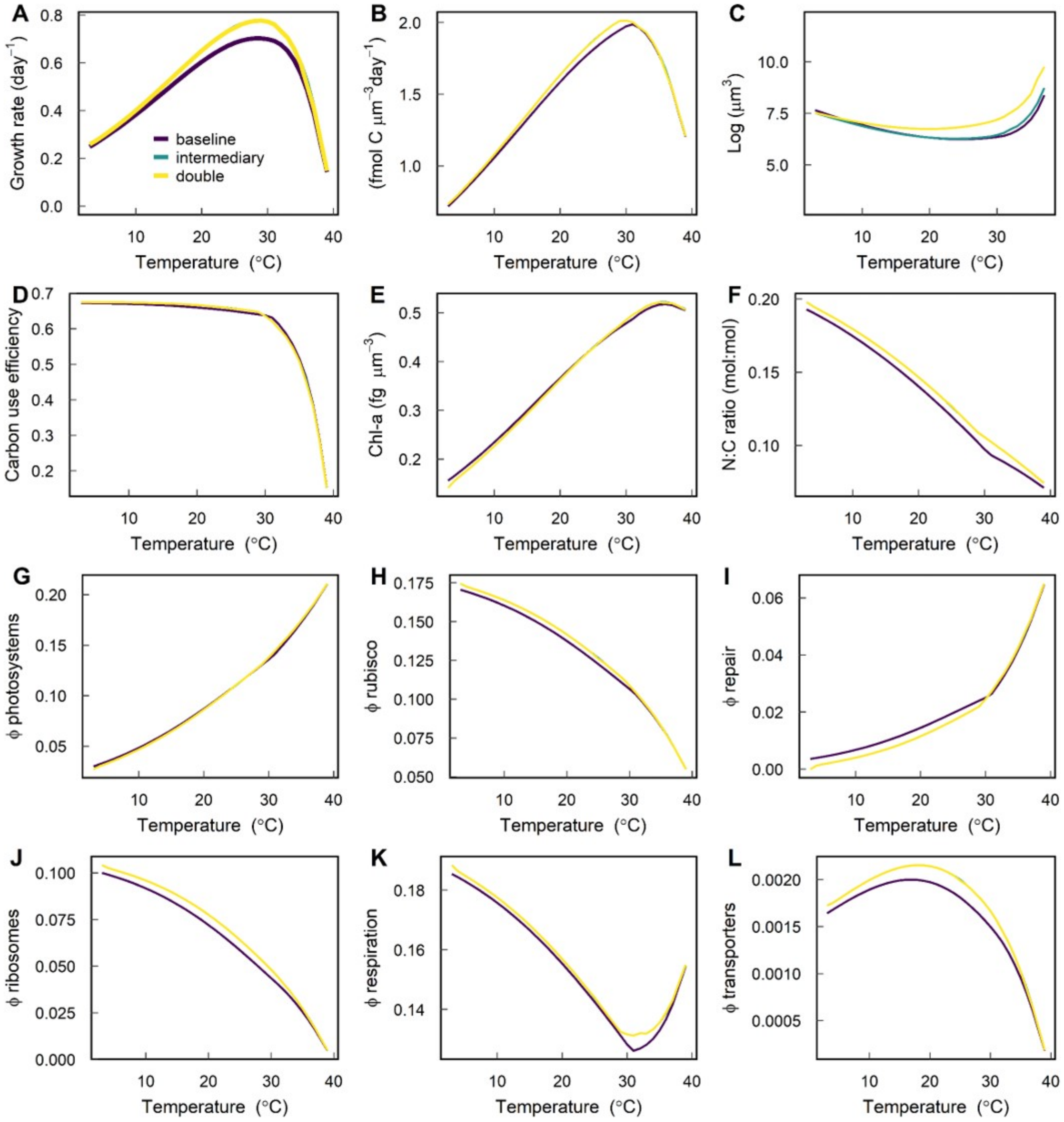

Figure S15. Sensitivity analysis to s_{lm}. This parameter sets the specific surface area of one lipid molecule in the membrane. Our baseline run (purple lines) assumed a constant value for s_{lm}. We then performed sensitivity analyses assuming that s_{lm} decreases as a function of temperature due to increases in the saturation level of lipids. s_{lm} was assumed to be 1.25 (intermediary; green lines) and 2 (double; yellow lines) times higher at the minimum temperature relative to the maximum temperature in our simulations.

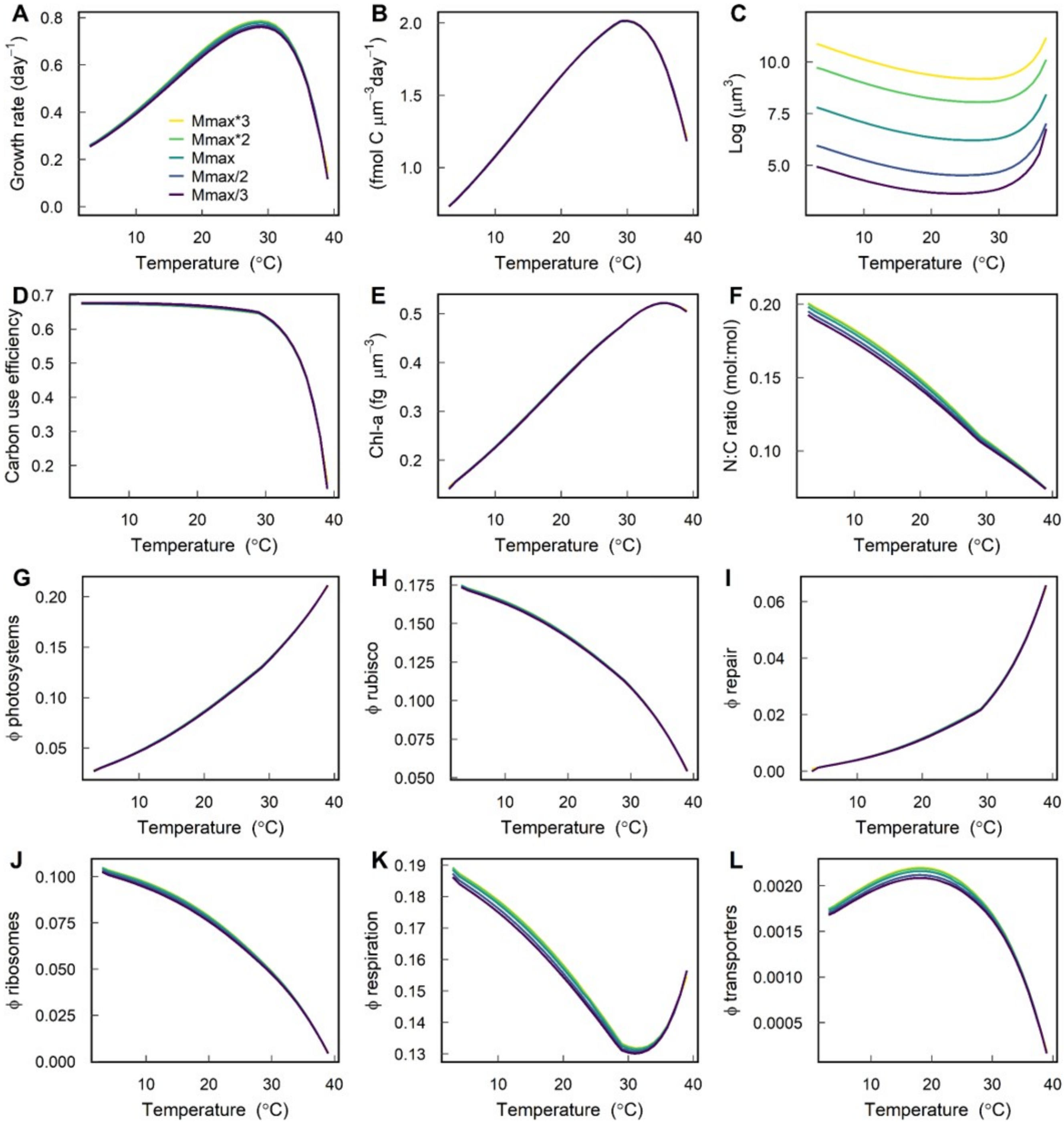

Figure S16. Sensitivity analysis to M_{max}. This parameter sets the maximum transporter to lipid ratio in the membrane. ϕ indicates relative proteome allocation in a given protein.

Tables

Table S1. Optimized parameters. Abbreviations, descriptions and units for the optimized variables. State variables correspond to the concentration of proteins and other macromolecules inside a phytoplankton cell.

	**State Variables**	
	Proteins	
p_p	Photosystems	molecules of p_p μm^{-3}
p_{ru}	rubisco	molecules of p_{ru} μm^{-3}
p_{tr}	Transporters	molecules of p_{tr} μm^{-3}
p_{ri}	Ribosomes	molecules of p_{ri} μm^{-3}
p_{gl}	Glycolysis	molecules of p_{gl} μm^{-3}
p_{lb}	Lipid Synthesis Pathway	molecules of p_{lb} μm^{-3}
p_{ld}	Lipid Degradation Pathway	molecules of p_{ld} μm^{-3}
p_{re}	Repair Proteins	molecules of p_{re} μm^{-3}
	Other macromolecules	
c_{ic}	Internal Carbon	molecules of C μm^{-3}
c_{in}	Internal Nitrogen	molecules of N μm^{-3}
c_{lm}	Membrane Lipids	molecules of c_{lm} μm^{-3}
c_{tag}	Lipid Storage	molecules of c_{tag} μm^{-3}
	**Parameters**	
	Relative proteome investments in proteins	
ϕ_p	Photosystems	unitless
ϕ_{ru}	rubisco	unitless
ϕ_{tr}	Transporters	unitless
ϕ_{ri}	Ribosomes	unitless
ϕ_{gl}	Glycolysis	unitless
ϕ_{lb}	Lipid Synthesis Pathway	unitless
ϕ_{ld}	Lipid Degradation Pathway	unitless
ϕ_{re}	Repair Proteins	unitless
	Other parameters	
β	Volume to Surface Area ratio of the cell	μm
α_{lm}	Fraction of lipid synthesis flux used to build membrane lipids	unitless
α_{ld}	Fraction of lipid synthesis flux used to fuel lipid degradation	unitless
α_{tag}	Fraction of lipid synthesis flux used to build lipid storage	unitless

Table S2. Parameter values. Abbreviations, descriptions, values, units and references. The values of tunable parameters are given in Table S3. All other parameter values were kept constants across our simulations. See Supplementary Text for more details regarding the source of the parameter values.

**Maximum reference turnover rates (# molecules per protein per time)**				
k_{ref_p}	Photosystems	7800	photons min^{-1}	(*51*)
$k_{ref_{ru}}$	rubisco	157	molecules of C min^{-1}	(*64*)
$k_{ref_{tr}}$	Transporters	120	molecules of N min^{-1}	
$k_{ref_{ri}}$	Ribosomes	114	molecules of aa min^{-1}	(*53*)
$k_{ref_{gl}}$	Glycolysis	120	molecules of C min^{-1}	BRENDA:EC2.7.1.11
$k_{ref_{lb}}$	Lipid Synthesis	120	molecules of C min^{-1}	BRENDA:EC6.4.1.2
$k_{ref_{ld}}$	Lipid Degradation	120	molecules of C min^{-1}	BRENDA:EC1.1.1.35
k_{ref_d}	Heat damage	Table S3	molecules of p min^{-1}	(*57*)
$k_{ref_{re}}$	Repair Proteins	1000	molecules of p min^{-1}	(*26*)
**Half saturation constants**				
K_p	Photosystems	1	μmol photon m^{-2} s^{-1}	
K_{ru}	rubisco	1	μM DIC	(*64*)
K_{tr}	Transporters	1	μM DIN	(*82*)
K_{in}	Nitrogen Metabolism	10000	molecules of N μm^{-3}	
K_{ic}	Carbon Metabolism	10000	molecules of C μm^{-3}	
K_{gl}	Glycolysis	10000	molecules of C μm^{-3}	
K_{lb}	Lipid Synthesis	10000	molecules of C μm^{-3}	
K_{ld}	Lipid Degradation	10000	molecules of C μm^{-3}	
**Temperature effects**				
E_a	Activation energy	Table S3	eV	(*10*)
$E_{a_{tr}}$	E_a of transporters	Table S3	eV	
E_d	Deactivation energy	Table S3	eV	(*10*)
T_d	Stress temperature	Table S3	$°C$	(*13*)
T_{ref}	Reference temperature	20	$°C$	
R	Boltzmann's constant	8.62×10^{-5}	eV/K	
**Space and density constraints**				
s_{tr}	Transporter surface area	1.26×10^{-5}	μm^2	(*72*)
s_{lm}	Lipid surface area	0.5×10^{-6}	μm^2	(*73*)
M_{max}	Transporter to lipid ratio	Table S3	unitless	
V_p	Photosystem volume	3.0×10^{-5}	μm^3	(*61*)
V_{li}	Lipid volume	1.5×10^{-9}	μm^3	
D_{max}	Maximum density	Table S3	Da μm^{-3}	(*29*)
$c_{tag_{max}}$	Maximum lipid storage	Table S3	molecules of lipid μm^{-3}	(*74*)
**Molecular weights**				
η_p	Photosystems	9082	aa/molecule of p	UniProt database
η_{ru}	rubisco	5933	aa/molecule of ru	UniProt database
η_{tr}	Transporters	1046	aa/molecule of tr	UniProt database
η_{ri}	Ribosomes	19393	aa/molecule of ri	UniProt database
η_{gl}	Glycolysis	15220	aa/molecule of gl	UniProt database
η_{lb}	Lipid Synthesis	13742	aa/molecule of lb	UniProt database
η_{ld}	Lipid Degradation	5128	aa/molecule of ld	UniProt database
η_{re}	Repair Proteins	6350	aa/molecule of re	UniProt database
η_{in}	Internal Nitrogen	14	Da	
η_{ic}	Internal Carbon	12	Da	
η_{aa}	Amino Acid	110	Da	
η_{gu}	Glucose	180	Da	
η_{li}	Lipid Storage	800	Da	(*76*)

**Carbon and Nitrogen quotas**				
η_{aac}	Amino Acids Carbon	5	C/aa	
η_{guc}	Glucose Carbon	6	C/molecule of gu	
η_{lic}	Lipids Carbon	16	C/molecule of li	(*15*)
q_{pt}	Protein quota	Table S3	N/C	(*49*)
q_p	Photosystems quota	0.10	N/C	(*49*)
q_{ri}	Ribosomes quota	0.33	N/C	(*49*)
**Energy conversion factors**				
e_p	Photosystems	1.0	ATP/photon	(*77*)
e_{ru}	rubisco	10	ATP/C	(*31*)
e_{tr}	Transporters	1.0	ATP/N	(*53*)
e_{ri}	Protein synthesis	48	ATP/aa	(*78*)
e_{gl}	Glycolysis	5.0	ATP/C	(*31*)
e_{lb}	Lipid Synthesis	3.9	ATP/C	(*31*)
e_{ld}	Lipid Degradation	6.6	ATP/C	(*31*)
e_{re}	Repair Proteins	4.5	ATP/photosystem	(*79*)
e_d	Heat damage	Table S3	ATP/photosystem	

Table S3. Tuned parameters. Parameter values tuned to fit the models to the different datasets. The model ID and corresponding dataset are as following: Schaum22 (*13*), Liang (*29*), O'Donnell (*14*), Schaum26 (*13*), Schaum32 (*13*), and Cyano (*27, 42, 81*). In addition to these, the k_{ref} values were increased in 50% and 30% and decreased in 40% relative to the original values reported in Table S2 to fit the model to the Liang, O'Donnell, and Cyano datasets, respectively. The parameter values fitted to simulate warm-evolved strains (i.e. Schaum26 and Schaum32) were E_a, $c_{tag_{max}}$, and the k_{ref} values which were decreased in 50% relative to the original values reported in Table S2 to fit the model to the Schaum32 dataset.

Parameter	Schaum22	Liang	O'Donnell	Schaum26	Schaum32	Cyano	Unit
E_a	0.5	1.0	0.8	1.2	1.4	1.5	eV
$E_{a_{tr}}$	$\frac{E_a}{2}$	$\frac{E_a}{2}$	$\frac{E_a}{2}$	$\frac{E_a}{2}$	$\frac{E_a}{2}$	$\frac{E_a}{2}$	eV
E_d	1.5	2.4	2.0	1.5	1.5	1.5	eV
T_d	43	31	32	43	43	36	°C
k_{ref_d}	1000	500	500	1000	1000	1000	p_p molecules min^{-1}
D_{max}	1.8×10^{10}	2.5×10^{11}	2.0×10^{11}	1.8×10^{10}	1.8×10^{10}	2.0×10^{11}	Da μm^{-3}
M_{max}	0.00045	0.0035	0.00045	0.00045	0.00045	0.0004	unitless
q_{pt}	0.20	0.20	0.25	0.20	0.20	0.20	mol N mol C^{-1}
$c_{tag_{max}}$	2.0×10^7	2.0×10^7	2.0×10^7	2.0×10^7	4.0×10^7	2.0×10^7	c_{tag} molecules μm^{-3}
e_d	$\frac{9\times10^7}{c_{tag_{max}}}$	$\frac{9\times10^7}{c_{tag_{max}}}$	$\frac{9\times10^7}{c_{tag_{max}}}$	$\frac{9\times10^7}{c_{tag_{max}}}$	$\frac{9\times10^7}{c_{tag_{max}}}$	$\frac{9\times10^7}{c_{tag_{max}}}$	energy per p_p
V_p	3×10^{-5}	3×10^{-5}	3×10^{-5}	3×10^{-5}	3×10^{-5}	1.5×10^{-5}	μm^3

Table S4. Tuned parameters for the lipid degradation simulations. Parameter values tuned to fit the model to the different datasets used in Figure S1 were the same as in Table S3 unless reported here.

Parameter	Schaum22	Liang	O'Donnell	Unit
D_{max}	1.8×10^{10}	2.5×10^{11}	2.0×10^{11}	Da μm^{-3}
M_{max}	0.00045	0.0035	0.00045	unitless

Table S5. Original source for the datasets used to validate simulations. The data described here was used in Figures 2, S1, and S2.

Model	Trait	Strain	Original Source
Cyano	growth rate	*Prochlorococcus marina* MIT9301	Figure 1 (*27*)
Cyano	cell size	*Prochlorococcus marina* MIT9301	Figure 1 (*27*)
Cyano	N:C quota	*Prochlorococcus* VOL7	Figure 4 (*81*)
Cyano	chlorophyll content	*Prochlorococcus* CCMP1986	Table 2 (*42*)
Liang	growth rate	*Chaetoceros* CCMP160	Figure 1 (*29*)
Liang	cell size	*Chaetoceros* CCMP160	Table 2 (*29*)
Liang	chlorophyll	*Chaetoceros* CCMP160	Table 2 (*29*)
O'Donnell	N:C quota	*Thalassiosira pseudonana* CCMP1335	Figure 3 (*14*)
Schaum	growth rates	*Thalassiosira pseudonana* CCMP1335	Figures 1 and 2 (*13*)
Schaum	carbon fixation rates	*Thalassiosira pseudonana* CCMP1335	Figures 1 and 2 (*13*)
Schaum	carbon use efficiency	*Thalassiosira pseudonana* CCMP1335	Figures 1 and 2 (*13*)
Schaum	cell size	*Thalassiosira pseudonana* CCMP1335	Figure 3 (*13*)
Schaum	chlorophyll	*Thalassiosira pseudonana* CCMP1335	Figure 3 (*13*)
Schaum	N:C quota	*Thalassiosira pseudonana* CCMP1335	Figure 3 (*13*)

Table S6. UniProt data for *Fragilariopsis cylindrus* on photosystems (η_p)

Entry	Protein Name	Da
A0A5Q0TZT6	Cytochrome b559 subunit alpha	10,888
A0A5Q0TZS1	Cytochrome b6	23,879
A0A5Q0TZS5	Cytochrome b6-f complex subunit 4	17,689
A0A5Q0TZW9	Cytochrome c-550 (Cytochrome c550)	17,695
A0A1E7EP28	Cytochrome c-553 (Cytochrome c553)	13,450
A0A5Q0TZT4	Cytochrome f	34,176
A0A1E7FAF2	Extrinsic protein in photosystem II	19,246
A0A1E7FRW8	Mog1p/PsbP-like protein	27,314
A0A1E7FPI4	Oxygen-evolving enhancer protein 1	32,694
A0A1E7FQE4	Photosystem I accessory protein	12,040
A0A5Q0U0A6	Photosystem I assembly protein Ycf3	19,335
A0A5Q0TZV3	Photosystem I assembly protein Ycf4	20,427
A0A5Q0TZN4	Photosystem I iron-sulfur center	8,798
A0A5Q0TZN1	Photosystem I P700 chlorophyll a apoprotein A1	83,615
A0A5Q0TZN0	Photosystem I P700 chlorophyll a apoprotein A2	81,784
A0A5Q0TZT1	Photosystem I reaction center subunit II	15,486
A0A5Q0TZS9	Photosystem I reaction center subunit III, PSI-F	20,690
A0A1E7FR70	Photosystem I reaction center subunit VIII	13,702
A0A5Q0TZU4	Photosystem I reaction center subunit XI	16,245
B2BHZ1	Photosystem II CP43 reaction center protein	42,798
A0A5Q0TZR8	Photosystem II CP43' reaction center protein	51,832
A0A5Q0TZW2	Photosystem II CP47 reaction center protein	56,255
A0A5Q0TZR0	Photosystem II D2 protein, PSII D2 protein	39,078
A0A1E7F2C7	Photosystem II reaction center M protein	11,772
A0A5Q0TZU8	Photosystem II reaction center protein H, PSII-H	7,359
A0A5Q0TZT0	Photosystem II reaction center protein Z	6,458
A0A1E7FXV0	Photosystem II reaction center Psb28 protein	13,381
A0A5Q0TZM8	Photosystem II reaction center PsbW protein	13,153
A0A1E7FQY0	Photosystem II stability/assembly factor	44,264
A0A1E7FNI8	PS II complex 12 kDa extrinsic protein 1	16,306
A0A1E7FMJ3	PS II complex 12 kDa extrinsic protein 2	7,561
A0A1E7FR83	PsbP C-terminal domain-containing protein 1	26,027
A0A1E7FSP5	PsbP C-terminal domain-containing protein 2	33,210
A0A1E7FGE2	PsbP C-terminal domain-containing protein 3	16,877
A0A1E7FKP9	Uncharacterized protein 1	31,732
A0A1E7FWC3	Uncharacterized protein 2	20,183
A0A1E7FC71	Uncharacterized protein 3	13,749
A0A1E7ETC2	Uncharacterized protein 4	20,618
A0A1E7ESH7	Uncharacterized protein 5	37,259
Total	All	999,025

Table S7. UniProt data for *Fragilariopsis cylindrus* on rubisco (η_{ru})

Entry	Protein Name	Da
A0A5Q0TZT8	Ribulose bisphosphate carboxylase large chain 1	54,047
A9XI12	Ribulose bisphosphate carboxylase large chain 2	53,298
C6FFZ0	Ribulose bisphosphate carboxylase large chain 3	50,134
A0A1E7ETJ3	Ribulose-1,5-bisphosphate carboxylase large subunit 4	65,991
E9MZ74	Ribulose-1,5-bisphosphate carboxylase/oxygenase large subunit 5	27,369
A0A5Q0TZZ3	Ribulose-1,5-bisphosphate carboxylase/oxygenase small subunit 1	12,266
A0A1E7FJ75	Ribulose-1,5-bisphosphate caboxylase, small subunit 2	66,077
A0A1E7FU72	Ribulose-1,5 bisphosphate carboxylase/oxygenase N-methyltransferase	61,979
A0A5Q0TZM0	Probable Rubisco transcriptional regulator	29,896
A0A679L9E8	Ribulose-bisphosphate carboxylase 1	24,835
A0A679L9E0	Ribulose-bisphosphate carboxylase 2	24,803
A0A679L928	Ribulose-bisphosphate carboxylase 3	24,877
G8GL28	Ribulose-bisphosphate carboxylase 4	20,127
A0A1E7FB03	Rubisco accumulation factor 1 C-terminal domain-containing protein	23,631
A0A5Q0TZU3	Rubisco expression protein cbbX	32,678
A0A1E7FP55	Ribulose-1,5-bisphosphate carboxylase-like protein rbcS2	72,119
A0A1E7EPL7	Uncharacterized protein	8,589
Total	All	652,716

Table S8. UniProt data for *Fragilariopsis cylindrus* on transporters (η_{tr})

Entry	Protein Name	Da
A0A1E7EM96	Nitrate/nitrite transporter 1	58,965
A0A1E7F1R4	Nitrate/nitrite transporter 2	56,084
Total	All	115,049

Table S9. UniProt data for *Fragilariopsis cylindrus* on ribosomes (η_{ri})

**Entry**	**Protein Name**	**Da**
A0A5Q0TZS3	30S ribosomal protein S2, chloroplastic	25,657
A0A5Q0TZR5	30S ribosomal protein S3, chloroplastic	24,077
A0A5Q0TZM2	30S ribosomal protein S4, chloroplastic	23,808
A0A5Q0TZQ2	30S ribosomal protein S5A, chloroplastic	18,710
A0A1E7EQX8	30S ribosomal protein S5B, chloroplastic	17,676
A0A5Q0TZM1	30S ribosomal protein S6, chloroplastic	11,731
A0A5Q0TZP2	30S ribosomal protein S7, chloroplastic	17,968
A0A5Q0TZX0	30S ribosomal protein S8, chloroplastic	15,747
A0A5Q0TZQ7	30S ribosomal protein S9, chloroplastic	15,091
A0A5Q0TZM9	30S ribosomal protein S10, chloroplastic	12,272
A0A5Q0TZQ0	30S ribosomal protein S11, chloroplastic	13,746
A0A5Q0TZQ9	30S ribosomal protein S12, chloroplastic	13,811
A0A5Q0TZR3	30S ribosomal protein S13, chloroplastic	14,053
A0A5Q0TZW1	30S ribosomal protein S14, chloroplastic	12,054
A0A1E7FWU8	30S ribosomal protein S15, chloroplastic	9,290
A0A5Q0TZL6	30S ribosomal protein S16A, chloroplastic	9,009
A0A1E7EJP8	30S ribosomal protein S16B, chloroplastic	8,823
A0A5Q0TZQ1	30S ribosomal protein S17A, chloroplastic	9,733
A0A1E7F266	30S ribosomal protein S17B, chloroplastic	9,064
A0A5Q0TZT7	30S ribosomal protein S18, chloroplastic	8,092
A0A5Q0U088	30S ribosomal protein S20, chloroplastic	11,427
A0A5Q0TZV0	50S ribosomal protein L1, chloroplastic	25,381
A0A5Q0TZQ3	50S ribosomal protein L2, chloroplastic	30,872
A0A5Q0TZP8	50S ribosomal protein L3, chloroplastic	23,646
A0A5Q0TZP3	50S ribosomal protein L4, chloroplastic	24,130
A0A5Q0U069	50S ribosomal protein L5A, chloroplastic	27,676
A0A1E7FKW4	50S ribosomal protein L5B, chloroplastic	20,115
A0A5Q0TZQ5	50S ribosomal protein L6, chloroplastic	19,768
A0A5Q0TZR1	50S ribosomal protein L24, chloroplastic	8,667
A0A5Q0TZW3	50S ribosomal protein L11, chloroplastic	14,867
A0A5Q0TZU5	50S ribosomal protein L12, chloroplastic	13,423
A0A5Q0TZP1	50S ribosomal protein L13, chloroplastic	17,306
A0A5Q0U080	50S ribosomal protein L14, chloroplastic	13,424
A0A5Q0TZP9	50S ribosomal protein L16, chloroplastic	15,505
A0A1E7FKR3	50S ribosomal protein L17	17,540
A0A5Q0TZP7	50S ribosomal protein L18, chloroplastic	15,409
A0A5Q0TZT2	50S ribosomal protein L19, chloroplastic	13,680
A0A5Q0TZP5	50S ribosomal protein L22, chloroplastic	13,138
A0A5Q0TZX3	50S ribosomal protein L23, chloroplastic	11,590
A0A5Q0TZV9	50S ribosomal protein L20, chloroplastic	13,220
A0A5Q0TZR2	50S ribosomal protein L29, chloroplastic	8,827
A0A5Q0TZS2	50S ribosomal protein L31	8,264
A0A5Q0TZM7	50S ribosomal protein L32, chloroplastic	6,395
A0A5Q0TZS6	50S ribosomal protein L33, chloroplastic	7,468
A0A5Q0TZX6	50S ribosomal protein L35, chloroplastic	7,512
A0A1E7EUK6	Eukaryotic translation initiation 1	27,279
A0A1E7EUK6	Eukaryotic translation initiation 2	27,279
A0A1E7EPZ2	Eukaryotic translation initiation factor 4C	15,928
A0A1E7FAG0	KRR1 small subunit processome	42,498
A0A1E7FFI9	rRNA biogenesis protein RRP36	45,348
A0A1E7F4X1	Translation initiation factor IF-1	17,147
A0A1E7F615	Ribosomal protein S5	25,530
A0A1E7FL01	Ribosomal protein S13	16,898
A0A1E7EU86	Ribosomal protein S17	14,248
A0A1E7FCS9	Ribosomal protein L19	21,994
A0A1E7FHF5	Uncharacterized protein	7,048

Table S10. UniProt data for *Fragilariopsis cylindrus* on ribosomes (η_{ri})

**Entry**	**Protein Name**	**Da**
A0A1E7FC77	40S ribosomal protein S1A	31,351
A0A1E7FC77	40S ribosomal protein S2A	31,351
A0A1E7F151	40S ribosomal protein S3A	29,504
A0A1E7F151	40S ribosomal protein S3B	29,504
A0A1E7FKC6	40S ribosomal protein S4	29,341
A0A1E7FU77	40S ribosomal protein S6	28,058
A0A1E7FKG7	40S ribosomal protein S7	21,281
A0A1E7EQ40	40S ribosomal protein S8	23,284
A0A1E7FSK6	40S ribosomal protein S9	16,014
A0A1E7EU35	40S ribosomal protein S11	18,786
A0A1E7FKK0	Putative 40S ribosomal protein S11	15,758
A0A1E7EV64	40S ribosomal protein S12	16,026
A0A1E7F4H0	Putative 40S ribosomal protein S19	17,583
A0A1E7F2P4	40S ribosomal protein S21	9,304
A0A1E7F2G5	40S ribosomal protein S23	15,705
A0A1E7FGN7	40S ribosomal protein S25	12,796
A0A1E7EXY9	40S ribosomal protein S26	12,240
A0A1E7FH54	Putative 40s ribosomal protein S27	9,208
A0A1E7F7Q1	40S ribosomal protein S30	7,478
A0A1E7F2B3	60S ribosomal export protein NMD3 1	47,840
A0A1E7F5D5	60S ribosomal export protein NMD3 2	15,679
A0A1E7FIQ9	60S ribosome subunit biogenesis processes	20,633
A0A1E7FRU4	Nucleolar GTP-binding protein 1	75,549
A0A1E7FFB1	60S ribosomal protein L5	35,278
A0A1E7FKE3	60S ribosomal protein L6 CgRPL6	19,713
A0A1E7FCE9	60S ribosomal protein L7A	29,013
A0A1E7FV69	60S ribosomal protein L7B	19,878
A0A1E7FGH8	60S ribosomal protein L7C	26,662
A0A1E7FQJ3	60S ribosomal protein L10a	25,570
A0A1E7FW47	60S ribosomal protein L12A	17,544
A0A1E7F2Q1	60S ribosomal protein L17	20,495
A0A1E7FWD8	60S ribosomal protein L18A	20,950
A0A1E7F5A7	60S ribosomal protein L18B	21,560
A0A1E7FII5	60S ribosomal protein L21	18,280
A0A1E7FH63	60S ribosomal protein L24	21,350
A0A1E7FEG2	60S ribosomal protein L27	22,654
A0A1E7FWC8	60S ribosomal protein L29	6,748
A0A1E7ER83	60S ribosomal protein L34	12,696
A0A1E7FV65	Putative 60S ribosomal protein RPL35	13,745
A0A1E7FQ84	60S ribosomal protein L36A	46,921
A0A1E7EQQ7	60S ribosomal protein L36B	11,958
A0A1E7F584	60S ribosomal protein L41	11,137
A0A1E7FG01	Ribosome biogenesis protein NSA2	29,805
A0A1E7FR96	Ribosome assembly factor mrt4	26,199
A0A1E7EVR0	Nucleolar GTP-binding protein 2	60,857
A0A1E7G0F3	Protein SDA1	96,797
A0A1E7F7J0	Receptor of activated protein kinase	35,286
Total	All	2,133,276

Table S11. UniProt data for *Fragilariopsis cylindrus* on glycolysis (η_{gl})

**Entry**	**Protein Name**	**Da**
A0A1E7FB10	Glyceraldehyde-3-phosphate dehydrogenase 1	36,050
A0A1E7EVR3	Glyceraldehyde-3-phosphate dehydrogenase 2A	40,224
A0A1E7F3G9	Glyceraldehyde-3-phosphate dehydrogenase 2B	40,256
A0A1E7F5I8	Glyceraldehyde-3-phosphate dehydrogenase 3	38,365
A0A1E7F6U1	Glyceraldehyde-3-phosphate dehydrogenase 4	35,353
A0A1E7FKZ8	Phosphopyruvate hydratase 1	46,465
A0A1E7FDE1	Phosphopyruvate hydratase 2	49,967
A0A1E7FI20	Phosphopyruvate hydratase 3	47,932
A0A1E7F743	Phosphoglycerate kinase 1	46,281
A0A1E7F6F1	Phosphoglycerate kinase 2A	45,715
A0A1E7FAA9	Phosphoglycerate kinase 2B	42,452
A0A1E7F9E3	Phosphoglycerate kinase 3	46,418
A0A1E7FTF5	Triosephosphate isomerase 1	28,696
A0A1E7FBC9	Triosephosphate isomerase 2	31,727
A0A1E7F1T3	Triosephosphate isomerase 3	37,090
A0A1E7F7T0	Triosephosphate isomerase 4	27,933
A0A1E7FCM0	Triosephosphate isomerase 5	16,219
A0A1E7FU26	Glucose-6-phosphate isomerase 2	90,368
A0A1E7FGC8	Glucose-6-phosphate isomerase 3	92,602
A0A1E7FTC8	Glucose-6-phosphate isomerase 4	62,047
A0A1E7FQL0	Pyruvate kinase 1	65,985
A0A1E7FM47	Pyruvate kinase 2	58,792
A0A1E7ETJ0	Pyruvate kinase 3A	62,267
A0A1E7FFD8	Pyruvate kinase 3B	62,035
A0A1E7FRL2	Pyruvate kinase 4	57,767
A0A1E7FJU7	Pyruvate kinase 5	54,003
A0A1E7EK67	Pyruvate kinase 6	27,415
A0A1E7F4U5	Pyruvate kinase 7	32,884
A0A1E7FJX6	Fructose-bisphosphate aldolase 1	43,605
A0A1E7FAR3	Fructose-bisphosphate aldolase 2	47,835
A0A1E7FVW8	Fructose-bisphosphate aldolase 3	43,380
A0A1E7FK31	Fructose-bisphosphate aldolase 4	39,220
A0A1E7F3F3	Fructose-bisphosphate aldolase 5	33,240
A0A1E7FQS7	Fructose-bisphosphate aldolase 6	39,713
A0A1E7G012	Phosphofructokinase ATP	57,279
A0A1E7F2K6	Phosphofructokinase PPi	46,659
Total	All	1,674,239

Table S12. UniProt data for *Fragilariopsis cylindrus* on lipid synthesis (η_{lb})

Entry	Protein Name	Da
A0A1E7FLT6	Very-long-chain (3R)-3-hydroxyacyl-CoA dehydrogenase 1	23,050
A0A1E7FRX0	Very-long-chain (3R)-3-hydroxyacyl-CoA dehydrogenase 2	83,049
A0A1E7FNT7	Acetyl-CoA carboxylase 1	224,242
A0A1E7EQN9	Acetyl-CoA carboxylase 2	253,211
A0A1E7FBY1	Protein xylosyltransferase 1	17,661
A0A1E7EVZ1	Protein xylosyltransferase 2	39,856
A0A1E7ESD6	N-acetylglucosaminylphosphatidylinositol	27,888
A0A1E7FRA1	GPI ethanolamine phosphate transferase	103,757
A0A1E7F7D9	GPI mannosyltransferase 1	49,775
A0A1E7FI90	GPI mannosyltransferase 2	76,055
A0A1E7END6	Phosphatidate cytidylyltransferase	32,857
A0A1E7F1U2	CDP-diacylglycerol–glycerol-3-phosphatidyltransferase	88,374
A0A1E7F492	CTP:phosphoethanolamine cytidylyltransferase 1	61,223
A0A1E7FQX8	CTP:phosphoethanolamine cytidylyltransferase 2	39,062
A0A1E7FJF5	Phosphatidate cytidylyltransferase 1	43,555
A0A1E7ETM0	Phosphatidate cytidylyltransferase 2	42,065
A0A1E7FDU5	Phosphatidate cytidylyltransferase 3	14,886
A0A1E7FCF1	CDP-diacylglycerol synthase	11,802
A0A1E7FUK9	3-ketoacyl-CoA synthase	53,568
A0A1E7FAH3	Uncharacterized protein 1	18,649
A0A1E7FWB6	Uncharacterized protein 2	29,500
A0A1E7FQT4	Uncharacterized protein 3	35,132
A0A1E7FVP0	Uncharacterized protein 4	56,745
A0A1E7FEM1	Uncharacterized protein 5	68,410
A0A1E7F544	Uncharacterized protein 6	17,255
Total	All	**1,511,627**

Table S13. UniProt data for *Fragilariopsis cylindrus* on lipid degradation (η_{ld})

Entry	Protein Name	Da
A0A1E7FKV7	Enoyl-CoA hydratase 1	84,089
A0A1E7FKK2	Enoyl-CoA hydratase 2	84,837
A0A1E7EUD0	Acyl-coenzyme A dehydrogenase	84,816
A0A1E7F0G4	Propanoyl-CoA:carbon dioxide ligase	81,184
A0A1E7FDF3	Phosphoinositide phospholipase C 1	91,020
A0A1E7EY21	Phosphoinositide phospholipase C 2	83,260
A0A1E7EJ58	Phosphoinositide phospholipase C 3	54,927
Total	All	**564,133**

Table S14. UniProt data for *Fragilariopsis cylindrus* on repair proteins (η_{re})

**Entry**	**Protein Name**	**Da**
A0A1E7FEE3	Non-specific serine/threonine protein	58,940
A0A5Q0TZM8	Photosystem II reaction center Psb2	13,153
A0A1E7F8G3	Protein phosphatase 1	30,228
A0A1E7EUJ0	Protein phosphatase 2	25,850
A0A1E7FHE3	Protein phosphatase 3	62,416
A0A1E7EY20	Peptidase M41 FtsH extracellular domain-containing protein	22,755
A0A1E7EQ26	ATP-dependent metalloprotease FtsH	67,184
A0A5Q0TZV2	ATP-dependent zinc metalloprotease	67,844
A0A5Q0TZN8	Protein translocase subunit SecY	47,483
A0A1E7EWW3	Replication factor-A protein 1 N-terminal domain-containing protein 1	55,099
A0A1E7FLZ8	Replication factor-A protein 1 N-terminal domain-containing protein 2	54,553
A0A1E7EIP7	Replication factor-A protein 1 N-terminal domain-containing protein 3	50,480
A0A5Q0TZN6	Chaperone protein DnaK	64,962
A0A5Q0TZN5	60 kDa chaperonin, chloroplastic	56,979
A0A1E7ETC2	Uncharacterized protein 1	20,618
Total	All	698,544

Table S15. Parameter values used for the sensitivity analysis of the lipid degradation pathway (Figure S8).

Parameter	baseline	decrease	increase	Unit
D_{max}	1.8×10^{10}	9×10^{9}	3.6×10^{10}	Da μm^{-3}
E_a	0.5	0.25	1.0	eV
e_{ld}	6.6	6.3	6.9	energy/C
f_{dr}	0.25	0.20	0.30	unitless
$k_{ref_{ld}}$	120	90	150	1/time
M_{max}	0.005	0.0025	0.01	unitless
